# Experimental and numerical investigation of rubberized concrete beam–column joints subjected to monotonic loading

**DOI:** 10.1038/s41598-026-67310-6

**Published:** 2026-08-25

**Authors:** Wafaa A. Salman, Ehab M. Lotfy, Ahmed M. Gomaa, Abdel-Rahman M. Naguib, Sally Hosny

**Affiliations:** 1 Civil Engineering Program, High Institute of Engineering and Technology, Arish, Egypt; 2 Department of Structural Engineering, Faculty of Engineering, Suez Canal University, Ismailia, Egypt; 3 Construction and Building Engineering Department, Faculty of Engineering and Technology, Egyptian Chinese University, Cairo, Egypt

**Keywords:** Rubberized concrete, Beam–column joints, Monotonic loading, Finite element analysis, Sustainable structures, Engineering, Materials science

## Abstract

The incorporation of waste tire rubber into structural concrete has attracted increasing attention as a sustainable approach for reducing environmental pollution while enhancing the deformation capacity and energy absorption of reinforced concrete (RC) members. However, the structural behavior of rubberized concrete beam–column joints remains insufficiently understood, particularly under monotonic loading conditions. This study presents an integrated experimental and numerical investigation of exterior RC beam–column joints incorporating crumb rubber as a partial replacement for fine aggregate. Four exterior beam–column joint specimens containing 0%, 10%, 15%, and 20% crumb rubber by volume were tested under monotonic lateral loading while maintaining a constant axial load on the column. The structural response was evaluated in terms of load–displacement behavior, ultimate load capacity, stiffness degradation, deformation capacity, energy absorption, crack development, and failure mode. A three-dimensional finite element model was developed using ABAQUS based on the Concrete Damage Plasticity (CDP) model and validated against the experimental results. The validated numerical model was subsequently employed to conduct a parametric study investigating the influence of axial load ratio on the structural performance of rubberized beam–column joints. The experimental results demonstrated that incorporating moderate amounts of crumb rubber significantly enhanced deformation capacity and energy absorption while maintaining satisfactory load-carrying capacity. The specimen containing 10% crumb rubber exhibited the best overall structural performance by providing the most balanced combination of strength, deformation capacity, and energy absorption, with only a limited reduction in ultimate strength compared with the control specimen. Numerical predictions showed excellent agreement with the experimental observations in terms of load–displacement response, cracking behavior, and failure mode. The parametric study further indicated that lower-to-moderate axial load ratios provided favorable structural performance, whereas excessive axial compression reduced the deformation capacity of the joints. The findings demonstrate that rubberized concrete can provide a practical and sustainable alternative for improving the overall structural performance of RC beam–column joints subjected to monotonic loading.

## Introduction

Beam–column joints are among the most critical regions in reinforced concrete (RC) frame structures because they transfer forces between beams and columns under combined shear, bending, and axial actions^1^. Unlike flexural members, stresses within the joint core are highly concentrated and strongly influenced by bond behavior, confinement, and diagonal compression strut action^1^. Joint failure is often sudden and brittle, particularly when transverse reinforcement is inadequate, and may lead to rapid stiffness loss or progressive collapse of moment-resisting frames^2^. Therefore, accurate evaluation of joint shear strength, stiffness retention, and deformation capacity remains a major challenge in structural engineering design^3^.

At the same time, the construction industry is increasingly seeking sustainable materials that reduce environmental impact without compromising structural safety. Waste tires represent a major environmental problem worldwide due to landfill demand, fire risk, and long-term ecological effects^4^. Recycling waste tires as crumb rubber for partial replacement of natural aggregates has emerged as a practical solution^5^. Rubberized concrete has shown beneficial properties such as higher deformability, improved impact resistance, vibration damping, crack control, and greater energy absorption^6^. These characteristics make it attractive for structural members where ductility and damage tolerance are required^7^. Recent experimental studies have further confirmed these advantages. Saad et al. demonstrated that incorporating 10% crumb rubber into reinforced concrete beams significantly enhanced impact resistance, ductility, and energy dissipation. Moreover, combining crumb rubber with steel fibers effectively compensated for the reduction in compressive strength, resulting in a balanced structural performance suitable for sustainable concrete applications^8^.

However, rubber incorporation also reduces compressive strength, elastic modulus, tensile strength, and bond quality because of the low stiffness of rubber particles and the weak interfacial transition zone with cement paste^9^. While these reductions may be acceptable in some noncritical applications, they become more significant in beam–column joints, where performance depends on confinement, anchorage, and stress transfer within a limited concrete core^10^. Therefore, conclusions obtained from beams, slabs, or pavements cannot be directly applied to joint regions^2^. Although many studies have examined rubberized concrete at the material level or in flexural members, research on beam–column joints remains limited^11^. Recent experimental and numerical studies have demonstrated that nonlinear finite element models can accurately predict the structural behavior of reinforced concrete beam–column joints^12^. Saad et al. developed validated ABAQUS models to investigate the influence of column axial load and CFRP strengthening on beam–column joint performance, showing excellent agreement with experimental results in terms of ultimate load, stiffness degradation, crack propagation, and failure modes. Similarly, finite element investigations on rubberized concrete members confirmed that the Concrete Damage Plasticity (CDP) model can effectively simulate the nonlinear response and damage evolution of rubberized reinforced concrete structures^13–15^.

Existing studies rarely combine experimental testing with validated three-dimensional nonlinear finite element analysis to comprehensively evaluate the behavior of rubberized reinforced concrete beam–column joints17. Consequently, reliable design recommendations for such structural systems remain unavailable^16–18^.

Another limitation is that current design provisions for RC joints were developed for conventional concrete and do not account for the modified behavior of rubberized concrete, including reduced stiffness, higher strain capacity, and altered confinement response^19^. Applying conventional equations directly to rubberized systems may therefore lead to conservative or inaccurate predictions. In addition, the combined influence of rubber ratio, axial load level, beam reinforcement, and joint transverse reinforcement has not been systematically quantified^20^.

Recent studies have significantly advanced the understanding of reinforced concrete beam–column joints through analytical modeling, experimental investigations, artificial intelligence techniques, and comprehensive review studies. Compression-field-based analytical models have been proposed to improve the prediction of shear strength in reinforced concrete slender beams without web reinforcement, providing a more rational understanding of shear transfer mechanisms and concrete compression fields^21^. In addition, experimental investigations on UHPC corner beam–column joints subjected to bi-directional cyclic loading demonstrated that advanced cementitious materials can significantly improve joint strength, ductility, and energy dissipation under complex loading conditions^22^.

Comprehensive studies have also identified the key structural parameters governing the cyclic behavior of reinforced concrete beam–column joints, including axial load ratio, reinforcement detailing, concrete compressive strength, joint confinement, and loading history, emphasizing their combined influence on joint performance and failure mechanisms^23^. More recently, artificial intelligence techniques have been introduced for structural assessment, where artificial neural network (ANN) models successfully predicted the joint shear strength of exterior beam–column joints using experimental databases, demonstrating excellent prediction accuracy and highlighting the potential of data-driven methods for structural engineering applications^24^. Furthermore, a recent state-of-the-art review summarized the structural behavior of hybrid reinforced concrete beam–column joints under cyclic loading, highlighting current advances, existing research limitations, and future directions for improving joint performance^25^. Collectively, these studies demonstrate the rapid advancement of analytical, experimental, numerical, and data-driven approaches for evaluating beam–column joints while highlighting the need for further investigation of sustainable rubberized concrete joints under monotonic loading.

Despite these important advances, previous studies have mainly focused on conventional or hybrid reinforced concrete joints subjected to cyclic loading, analytical shear prediction methods, or artificial intelligence-based strength estimation. However, the structural performance of rubberized reinforced concrete beam–column joints under monotonic loading has received very limited attention. Moreover, integrated investigations combining full-scale experimental testing with validated three-dimensional finite element modeling remain scarce. Therefore, the present study addresses these research gaps by experimentally and numerically evaluating the structural behavior of rubberized reinforced concrete beam–column joints incorporating different crumb rubber replacement ratios under monotonic loading.

To address these gaps, this study presents a combined experimental and numerical investigation of rubberized concrete beam–column joints under monotonic loading. Four one-third-scale exterior joint specimens with different crumb rubber replacement ratios were tested to evaluate load–displacement response, joint shear strength, stiffness degradation, crack propagation, ductility, energy absorption, and failure mode. A conventional concrete specimen without rubber replacement was used as a control specimen. In parallel, a three-dimensional nonlinear finite element model was developed in ABAQUS using the Concrete Damage Plasticity (CDP) model and validated against the experimental results. A subsequent parametric study examined the effects of axial load ratio, concrete compressive strength, beam longitudinal reinforcement ratio, and joint transverse reinforcement ratio.

Accordingly, the present study provides a comprehensive experimental and numerical investigation of rubberized reinforced concrete beam–column joints subjected to monotonic loading. Unlike previous investigations that mainly focused on conventional concrete, cyclic loading conditions, analytical shear models, or artificial intelligence prediction techniques, the present work integrates laboratory testing with validated three-dimensional finite element analysis to evaluate strength, stiffness, ductility, crack propagation, energy absorption, and failure mechanisms. The outcomes contribute to a better understanding of the structural performance of sustainable rubberized concrete beam–column joints and provide a basis for future analytical models and performance-based design recommendations.

## Experimental study

### Materials

The experimental program was designed to investigate the structural behavior of reinforced concrete beam–column joints incorporating rubberized concrete under monotonic loading. Four concrete mixtures were prepared by partially replacing natural fine aggregate with crumb rubber at replacement levels of 0%, 10%, 15%, and 20% by volume of sand, designated as CR-0, CR-10, CR-15, and CR-20, respectively.

#### Cement

Ordinary Portland Cement (OPC) complying with ASTM C150 Type I^26^ was used as the cementitious binder. A constant cement content of 350 kg/m^3^ was adopted for all concrete mixtures to ensure a consistent basis for evaluating the influence of crumb rubber incorporation.

#### Fine and coarse aggregates

Natural river sand was used as the fine aggregate, while crushed gravel with a nominal maximum size of 38 mm was used as the coarse aggregate at a constant dosage of 1272 kg/m^3^. The aggregates satisfied the grading requirements of ASTM C33^27^, and the gravel content was maintained constant throughout the experimental program.

#### Crumb rubber

**Fig. 1:**
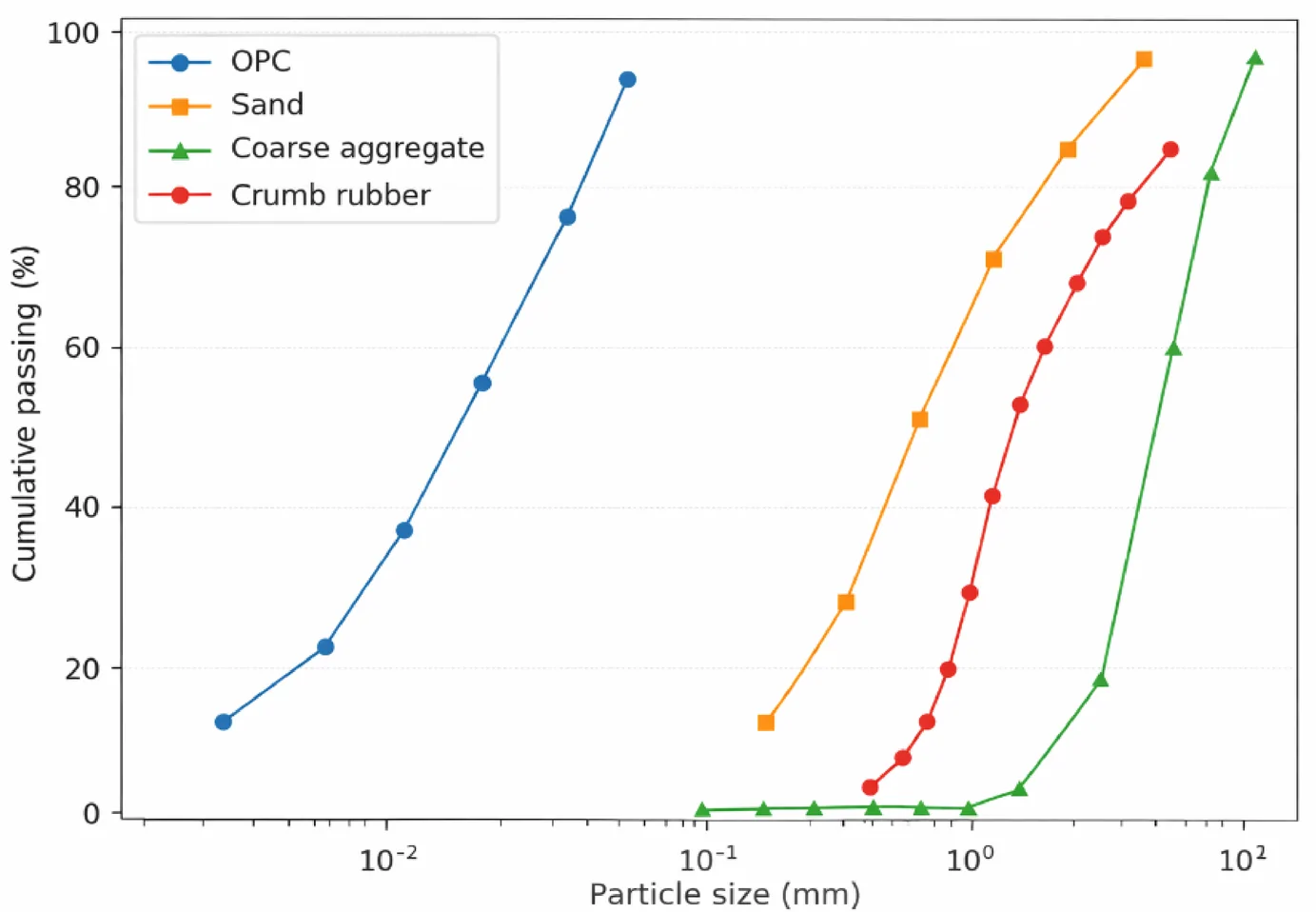
Particle size distribution (PSD) curves of OPC, natural fine aggregate, crushed coarse aggregate, and crumb rubber particles.

Crumb rubber obtained from mechanically shredded waste automobile tires was used as a partial replacement for natural fine aggregate. The crumb rubber particles were black in color with an irregular to sub-angular shape and a rough surface texture. The measured physical properties are summarized in Table 1. The crumb rubber had a specific gravity of 1.72, a bulk density of 0.83 g/cm^3^, a moisture content of 2%, and a fineness modulus of 4.48. The particle size distribution of the crumb rubber, together with those of the cement, natural sand, and coarse aggregate, is presented in Fig. 1. The crumb rubber was incorporated at replacement levels of 0%, 10%, 15%, and 20% by volume of natural fine aggregate while maintaining all other mixture proportions unchanged. Although the water absorption of the crumb rubber was not measured in the present study, previous investigations have reported typical values ranging between 4% and 5% after 24 h immersion, reflecting the relatively low water absorption compared with natural aggregates. The rough surface morphology contributes to mechanical interlocking with the surrounding cement paste; however, the hydrophobic nature and relatively low stiffness of rubber particles weaken the interfacial transition zone, leading to reduced stiffness and compressive strength while improving deformation capacity and energy absorption.

**Table 1:** Physical properties of crumb rubber.

Property	Value
Appearance	Black
Shape	Irregular to sub-angular
Surface texture	Rough
Specific gravity	1.72
Bulk density	0.83 g/cm^3^
Moisture content	2%
Fineness modulus	4.48
Water absorption	Not measured in the present study (typically 4–5% after 24 h immersion according to previous studies)

#### Mix proportions

The concrete mixtures were proportioned following the ACI 211.1^28^ mix design procedure for normal-weight concrete. The control mixture was designed to achieve a target 28-day compressive strength of approximately 40 MPa, which is representative of structural reinforced concrete commonly used in beam–column joints. A target workability corresponding to a medium slump suitable for reinforced concrete casting was adopted to ensure adequate compaction and proper placement of concrete around the congested reinforcement within the joint region. To isolate the influence of crumb rubber incorporation, the water-to-cement ratio (0.50), cement content, coarse aggregate content, and mixing procedure were maintained constant for all mixtures, while only the crumb rubber replacement ratio was varied.

All concrete mixtures were prepared with a constant water-to-cement ratio (w/c = 0.50) to ensure that the observed behavioral changes were primarily attributed to the incorporation of crumb rubber. The adopted concrete mix proportions are summarized in Table 2.

**Table 2:** Concrete mix proportions (kg/m^3^) with volumetric replacement of natural fine aggregate by crumb rubber.

Mix ID	Cement (kg/m^3^)	Water (kg/m^3^)	w/c	Gravel (kg/m^3^)	Sand (kg/m^3^)	Crumb rubber (kg/m^3^)	Rubber content (% by volume of sand)
CR-0	350	180	0.50	1272	636	0.0	0
CR-10	350	180	0.50	1272	612.3	23.7	10
CR-15	350	180	0.50	1272	600.4	35.6	15
CR-20	350	180	0.50	1272	588.5	47.5	20

#### Mechanical properties of concrete

The compressive strength and splitting tensile strength of the rubberized concrete mixtures were experimentally determined at the age of 28 days. The compressive strength was measured using 150 × 150 × 150 mm concrete cube specimens in accordance with BS EN 12390-3^29^, while the splitting tensile strength was determined following ASTM C496/C496M^30^.

The measured compressive strengths were 46.3, 42.0, 36.4, and 29.8 MPa for mixtures CR-0, CR-10, CR-15, and CR-20, respectively. The corresponding splitting tensile strengths were 3.65, 3.30, 3.05, and 2.75 MPa, respectively. As expected, increasing the crumb rubber replacement ratio resulted in a gradual reduction in both compressive and tensile strengths because of the lower stiffness of rubber particles and the weaker interfacial transition zone between the rubber particles and the cement matrix.

The reduction in mechanical properties is primarily attributed to the relatively low stiffness of crumb rubber and its weaker bond with the surrounding cement paste. Nevertheless, concrete mixtures containing up to 15% crumb rubber maintained acceptable mechanical properties while exhibiting enhanced deformation capacity, whereas the 20% replacement level represented the limiting condition with a more pronounced reduction in stiffness and strength.

#### Reinforcing steel

Two types of reinforcing steel bars supplied by Ezz Steel Company, Egypt, were used in the experimental program. The reinforcement bars complied with the requirements of the Egyptian Standard Specifications (E.S.S. 262/2011)^31^. Mild steel bars with a diameter of 8 mm were used as transverse reinforcement (stirrups), whereas high-strength deformed steel bars with a diameter of 12 mm were used as the longitudinal reinforcement of the beam–column joint specimens.

**Fig. 2:**
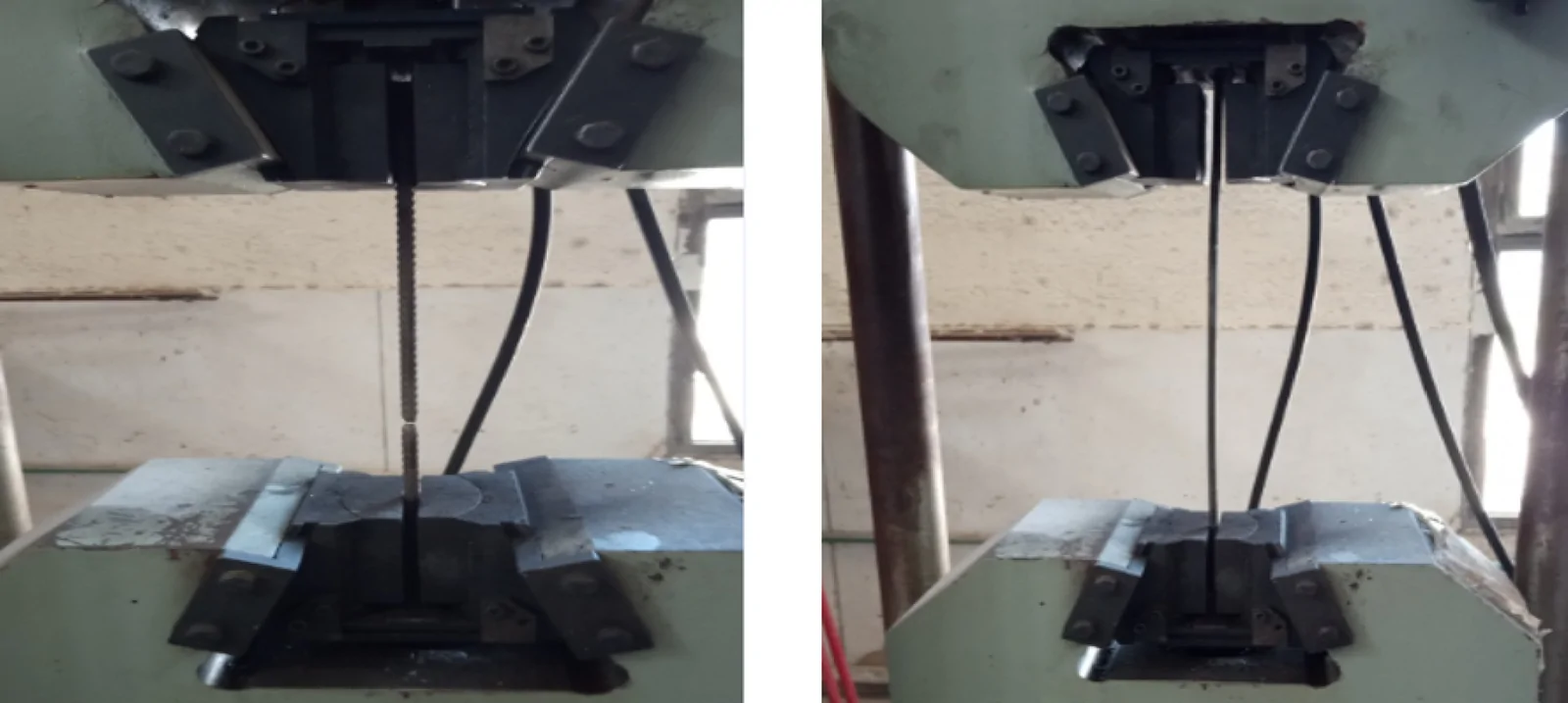
Experimental tensile stress–strain curves of the reinforcing steel bars.

Tensile tests were carried out to determine the mechanical properties of both reinforcement types. The measured yield strength (Fy) and ultimate tensile strength (Fu) are summarized in Table 3. The 8 mm mild steel bars exhibited a yield strength of 280 MPa and an ultimate tensile strength of 370 MPa, whereas the 12 mm high-strength deformed bars exhibited a yield strength of 420 MPa and an ultimate tensile strength of 570 MPa. The experimentally obtained stress–strain relationships, shown in Fig. 2, were adopted in both the experimental interpretation and the finite element modeling.

**Table 3:** Mechanical properties of reinforcing steel determined from tensile tests.

Reinforcement type	Diameter (mm)	Fy (MPa)	Fu (MPa)
Mild Steel	8	280	370
High-strength deformed steel	12	420	570

### Specimen details

A total of four one-third-scale reinforced concrete exterior beam–column joint specimens were fabricated and tested under monotonic loading in this experimental program. The study was designed to investigate the influence of crumb rubber incorporation on joint-level structural performance by varying the rubber replacement ratio while maintaining identical geometric dimensions, reinforcement detailing, and loading configuration across all specimens. This approach ensures that any observed variation in structural response can be directly attributed to changes in concrete composition rather than geometric or reinforcement-related factors.

The tested specimens represent one-third-scale exterior beam–column joints extracted from a typical reinforced concrete moment-resisting frame commonly employed in low- to mid-rise residential, educational, and office buildings. The prototype structure consists of conventional cast-in-place reinforced concrete frames designed according to current reinforced concrete design practice, where exterior joints are critical regions for transferring gravity and lateral loads between beams and columns. The one-third geometric scaling was adopted to satisfy the loading capacity and dimensional limitations of the available laboratory testing facilities while preserving the governing structural behavior of the prototype. Particular attention was given to maintaining the reinforcement detailing, member proportions, and joint geometry to ensure realistic stress transfer, crack development, and joint shear mechanisms during testing. Consequently, although reduced in size, the adopted specimens provide a representative simulation of the structural response of full-scale exterior beam–column joints subjected to monotonic loading.

**Fig. 3:**
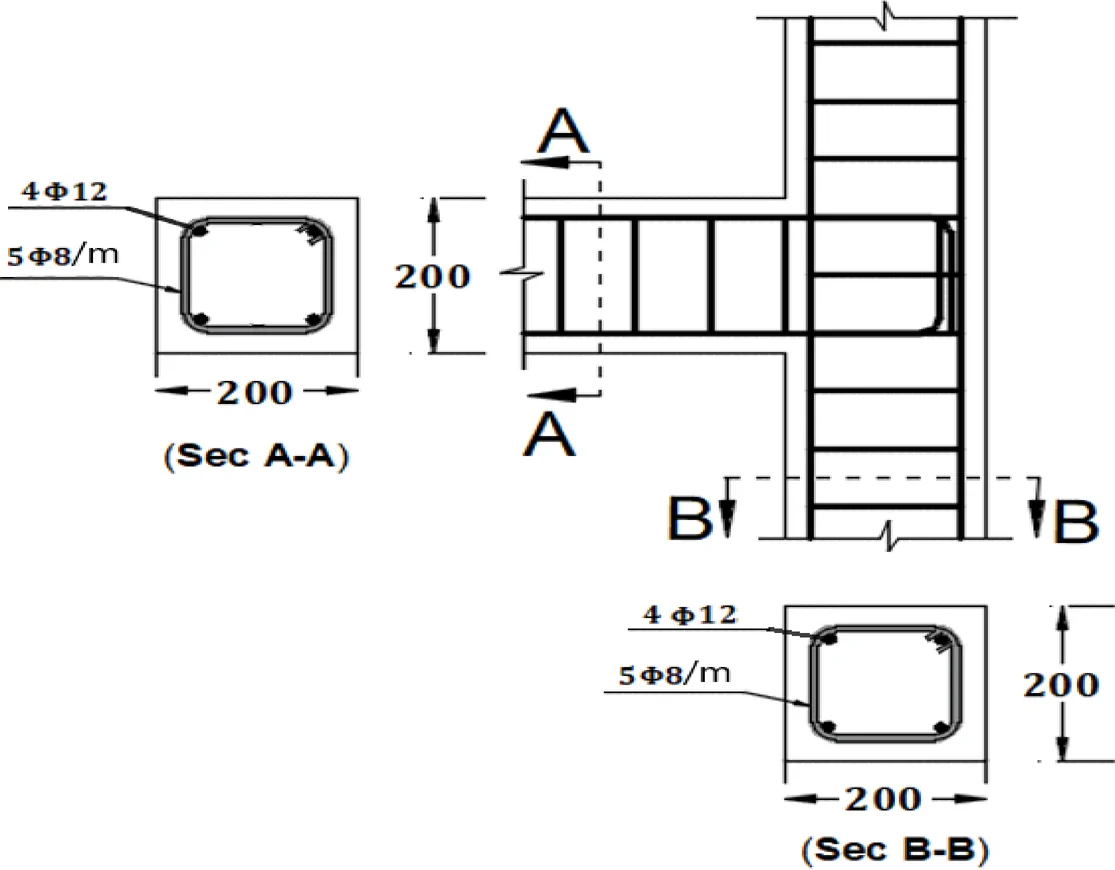
Typical reinforcement arrangements.

All specimens were constructed with identical dimensions. The column had a square cross-section of 200 × 200 mm and a total height of 1000 mm, while the beam had a cross-section of 200 × 200 mm and a cantilever length of 800 mm measured from the column face. These dimensions were selected to ensure realistic stress development within the joint core and to promote representative joint shear behavior under laboratory conditions. The general reinforcement configuration adopted for all specimens is schematically illustrated in Fig. 3.

The four specimens differed only in the percentage of crumb rubber used as partial replacement of fine aggregate. The control specimen, designated BCJ-CR0, was constructed using conventional concrete without rubber replacement and served as the reference for performance comparison. The remaining three specimens were constructed using rubberized concrete with 10%, 15%, and 20% volumetric replacement of sand, designated as BCJ-CR10, BCJ-CR15, and BCJ-CR20, respectively.

All beam–column joint specimens were reinforced with identical steel reinforcement to isolate the effect of rubber content on structural behavior. Longitudinal reinforcement in both the beam and column consisted of four 12 mm diameter deformed steel bars (4Ø12), arranged symmetrically to ensure adequate flexural capacity and anchorage within the joint region. Transverse reinforcement was provided using 8 mm diameter steel stirrups. In both beam and column regions, the stirrups were spaced at a density corresponding to 5Ø8 per meter. This uniform reinforcement arrangement was maintained across all specimens to ensure consistent confinement and shear resistance capacity independent of rubber content.

The reinforcement ratios were intentionally selected to prevent premature flexural failure of the beam, thereby promoting joint shear–controlled behavior under monotonic loading. By maintaining constant geometry and reinforcement detailing while varying only the rubber replacement ratio (0–20%), the experimental matrix enables a direct and systematic evaluation of the influence of rubber incorporation on joint shear strength, stiffness response, crack development, and failure characteristics.

### Casting and curing

All four beam–column joint specimens, along with companion standard cube and cylinder specimens for material characterization, were cast under controlled laboratory conditions to ensure uniformity of material properties and minimize variability between batches. Each concrete mixture corresponding to the designated rubber replacement ratio (CR-0, CR-10, CR-15, and CR-20) was prepared separately, while maintaining identical mixing procedures and environmental conditions.

The concrete was prepared using a conventional mechanical mixer. Initially, all dry constituents, including cement, sand, coarse aggregate, and crumb rubber (where applicable), were dry-mixed for approximately two minutes to ensure uniform distribution of rubber particles within the aggregate matrix. Special attention was given to the homogeneous dispersion of crumb rubber in order to prevent particle clustering and ensure consistent mechanical performance. Subsequently, the mixing water was added gradually while mixing continued for an additional three to four minutes to achieve a uniform and workable mixture. This staged mixing procedure was adopted to enhance coating of rubber particles by the cement paste and to reduce the risk of segregation or weak interfacial zones.

The fresh concrete was then placed into rigid wooden formwork containing the pre-installed longitudinal and transverse steel reinforcement. Plastic spacers were used to maintain the specified concrete cover and ensure accurate positioning of reinforcement within the joint region. Internal vibration was carefully applied to achieve proper compaction and eliminate entrapped air without causing segregation, particularly in mixtures containing higher rubber content.

**Fig. 4:**
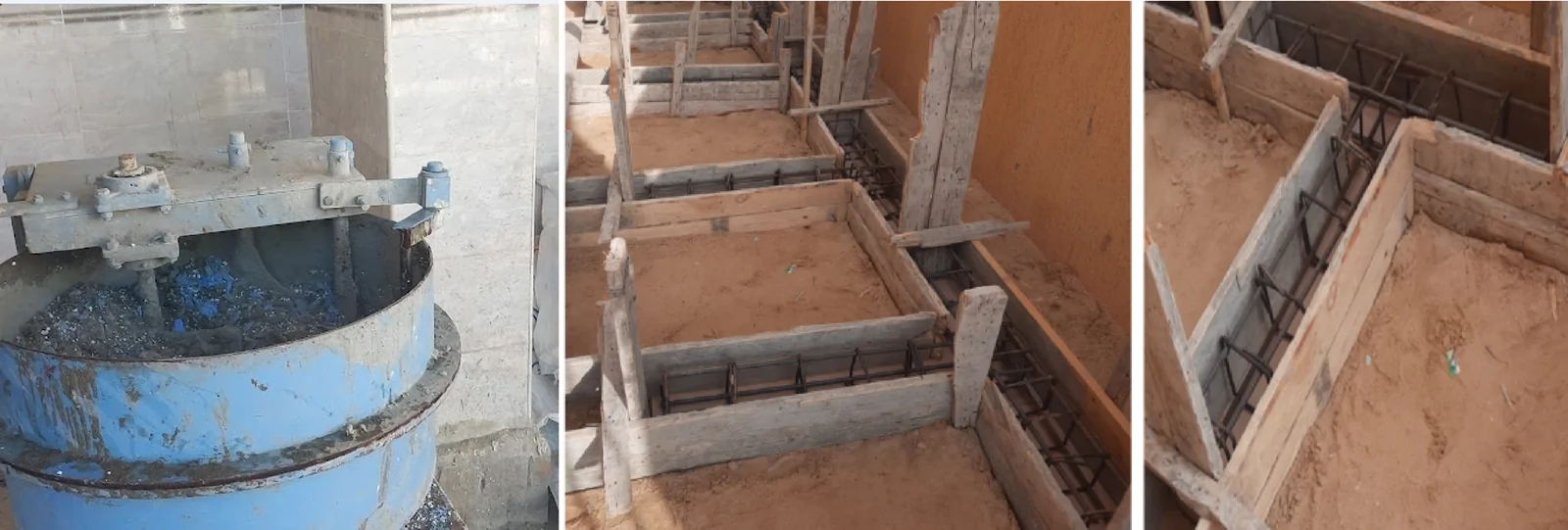
Mixing and casting procedure of rubberized concrete specimens.

After casting, all specimens were covered with plastic sheets to prevent moisture loss and were left undisturbed for 24 h prior to demolding. Following demolding, the specimens were cured by continuous water spraying until the testing age of 28 days, in accordance with the provisions of the Egyptian Code of Practice for the Design and Construction of Reinforced Concrete Structures (ECP 203–2020)^32^. The main stages of the concrete mixing, casting, and curing process are illustrated in Fig. 4.

### Test setup and loading procedure

All beam–column joint specimens were tested under monotonic lateral loading to evaluate their structural response in terms of load–displacement behavior, joint shear strength, stiffness characteristics, crack development, and failure mode. The experimental tests were conducted using a calibrated hydraulic loading system with a maximum capacity of 1000 kN, mounted on a rigid steel reaction frame to ensure stable boundary conditions during testing. The column base of each specimen was rigidly anchored to the strong floor using high-strength anchor bolts to simulate a fixed support condition. A constant axial compressive load was applied at the top of the column using a hydraulic jack to represent the effect of gravity loads in real structural systems. The axial load level was maintained constant throughout the test to replicate realistic stress conditions within the joint core. The applied axial compressive load corresponded to an axial load ratio of 0.10 (10% of the nominal axial compressive capacity of the column) and was maintained constant throughout all tests.

The monotonic loading protocol was intentionally adopted to investigate the fundamental structural behavior of rubberized concrete beam–column joints under static loading conditions. This loading scheme enables a clear evaluation of load-carrying capacity, stiffness degradation, deformation characteristics, crack propagation, energy absorption, and failure mechanisms without the additional complexities associated with load reversals. Therefore, the present study establishes a baseline understanding of the structural performance of rubberized beam–column joints under gravity-type loading. It should be noted that the findings of this investigation are limited to monotonic loading conditions and should not be directly extended to seismic applications, where cyclic loading, stiffness deterioration, cumulative damage, and bond degradation play a significant role.

Monotonic lateral load was applied at the free end of the beam through a hydraulic actuator acting horizontally. The load was transferred through a steel loading plate to ensure uniform stress distribution and to prevent local crushing at the loading point. The loading protocol was conducted under displacement-controlled conditions to ensure stable crack development and to capture post-peak response. The load was increased gradually at a slow rate to maintain quasi-static conditions and allow careful observation of crack initiation, propagation, and damage progression.

The monotonic loading tests were conducted using a servo-controlled MTS hydraulic testing machine operating under displacement-controlled conditions at a constant loading rate of 1 mm/min. The applied lateral load was measured using a calibrated 2000 kN MTS load cell with an accuracy of ± 0.5% of the applied load in accordance with ASTM E4/ISO 7500-1 Class 0.5^33^. The beam-end displacement was measured directly using the integrated MTS actuator displacement transducer (250 mm stroke), calibrated according to ASTM E2309^34^ with an accuracy of approximately ± 0.25% of the indicated displacement. Both load and displacement data were continuously recorded using the built-in digital data acquisition system of the MTS testing machine throughout the testing process, enabling continuous acquisition of the complete load–displacement response of each specimen until failure.

**Fig. 5:**
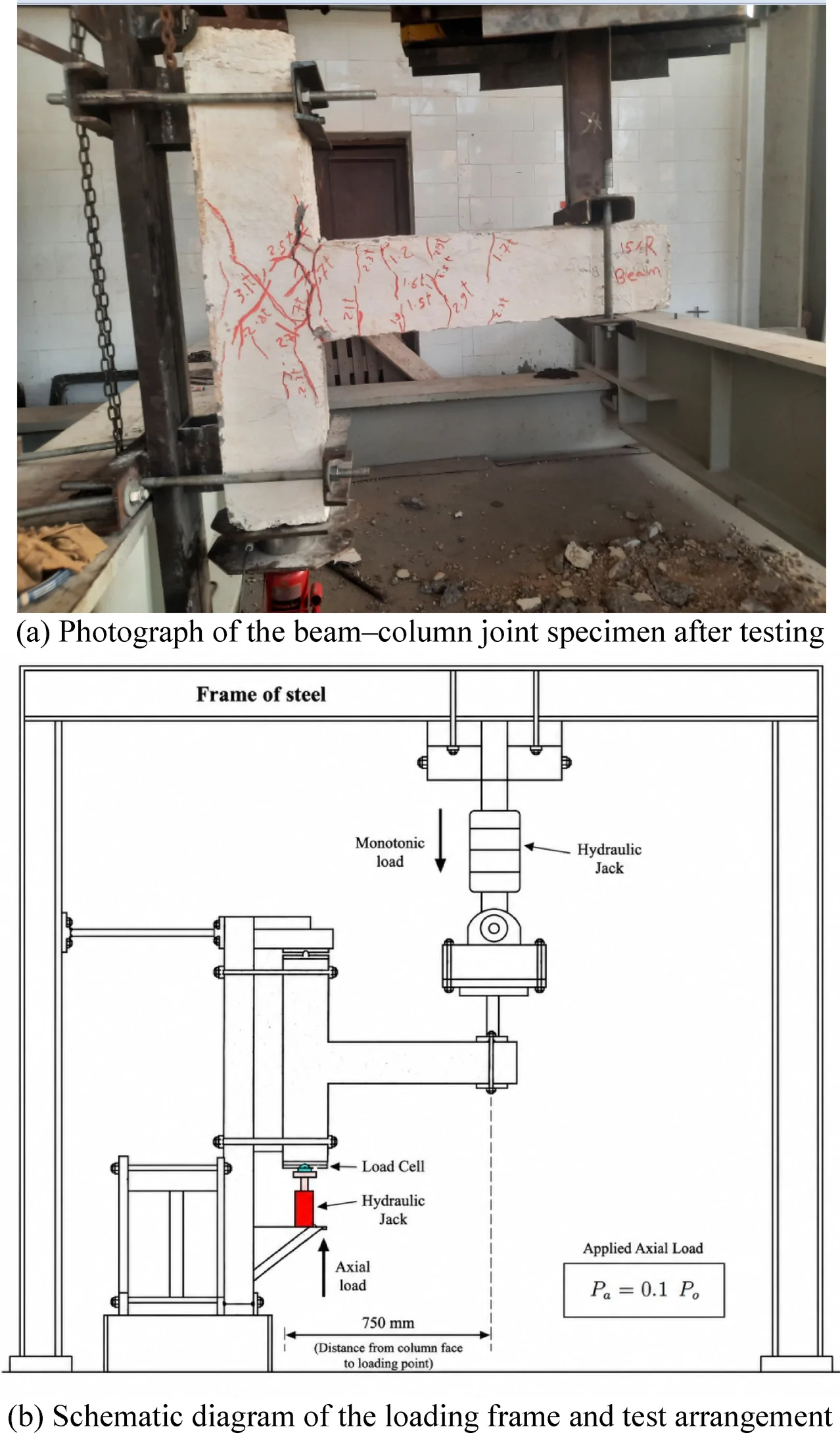
Experimental setup: (**a**) Photograph of the beam–column joint test setup; (**b**) Schematic diagram showing the loading arrangement, loading point, and net beam span.

During testing, crack patterns were marked manually on the concrete surface at regular load intervals. Particular attention was given to the joint core region to monitor diagonal cracking, bond deterioration, and confinement effectiveness. The test continued until significant strength degradation occurred or complete failure of the joint was observed. A schematic illustration of the adopted test setup, boundary conditions, axial load application, and lateral loading configuration is presented in Fig. 5.

Crack initiation and propagation were monitored by continuous visual observation during the testing process. At regular loading intervals, all visible cracks were manually marked on the concrete surface to document their initiation, propagation, and final crack patterns. Since the primary objective of this investigation was to evaluate the global structural response of the beam–column joints, reinforcement strain gauges and quantitative crack-width measurements were not included in the experimental program. Consequently, the discussion of cracking behavior is based on visual observations, crack distribution, and failure modes rather than quantitative crack-width measurements.

## Experimental result

### Load–deflection response and energy absorption

**Fig. 6:**
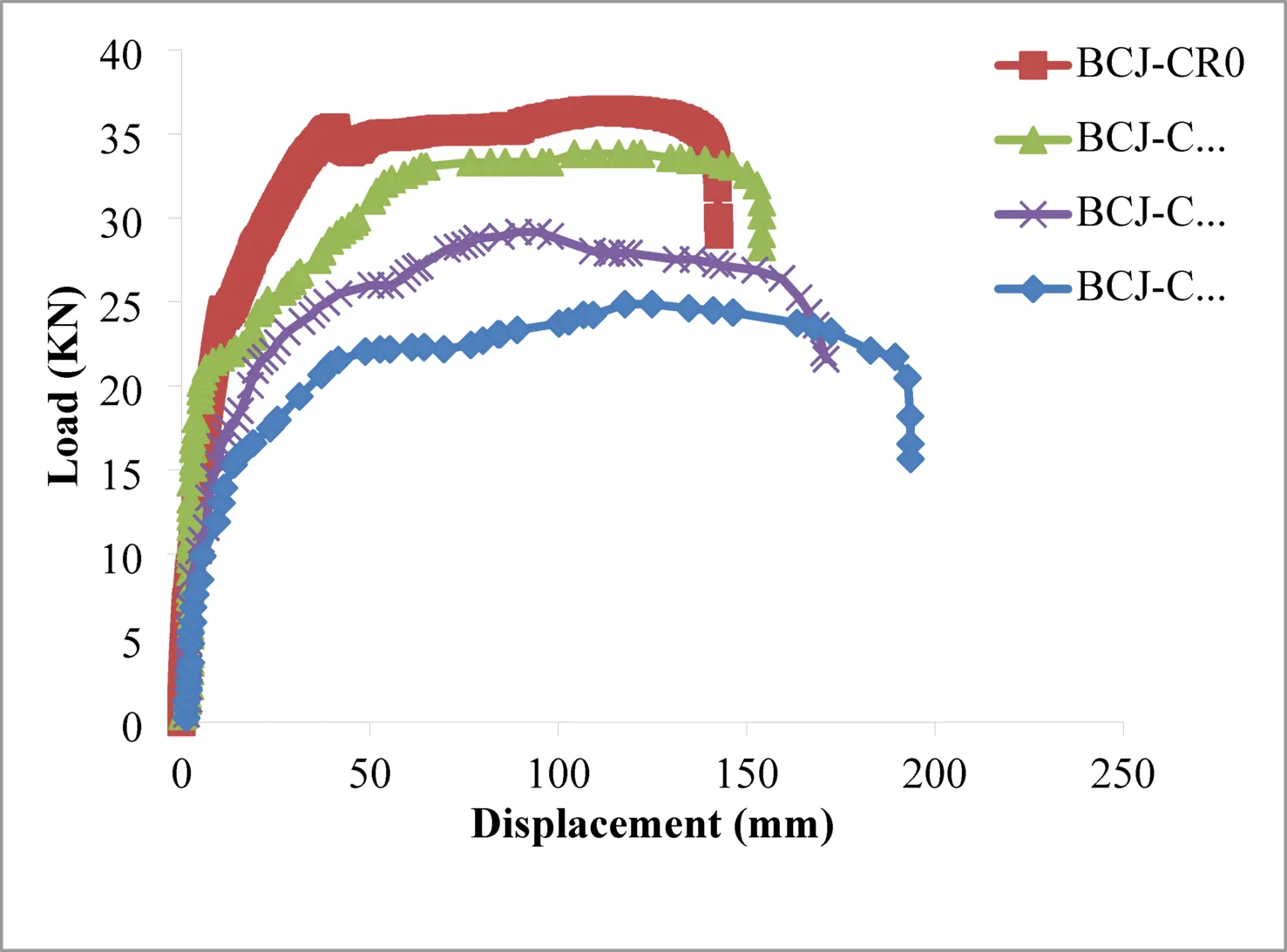
Load–Displacement curves of the tested beam specimens.

The response curves presented in Fig. 6 represent the lateral load–displacement relationship of the tested beam–column joint specimens. The applied lateral load was directly measured by the servo-controlled hydraulic testing machine, while the corresponding beam displacement was automatically recorded by the machine’s built-in digital data acquisition system.

As shown in Fig. 6, the control specimen (BCJ–CR0) exhibited the highest load-carrying capacity, reaching an ultimate load of 36.5 kN at a displacement of approximately 140 mm. The response was characterized by relatively high initial stiffness followed by a limited post-peak deformation capacity, indicating a comparatively brittle structural response. With increasing crumb rubber content, the ultimate load gradually decreased to 33.5 kN, 29.0 kN, and 25.0 kN for BCJ–CR10, BCJ–CR15, and BCJ–CR20, respectively. This reduction is primarily attributed to the lower stiffness of crumb rubber and the weaker interfacial bond between the rubber particles and the surrounding cementitious matrix.

From a micromechanical perspective, the lower elastic modulus of crumb rubber reduces the stiffness of the cementitious matrix and weakens the interfacial transition zone (ITZ), leading to earlier crack initiation and lower load-carrying capacity. However, the elastic nature of rubber particles enables greater strain accommodation and delays crack localization, thereby improving deformation capacity and energy absorption.

Despite the reduction in strength, the incorporation of crumb rubber significantly enhanced the deformation capacity of the specimens. The ultimate displacement increased from 140 mm for the control specimen to 160 mm, 170 mm, and 190 mm for BCJ–CR10, BCJ–CR15, and BCJ–CR20, respectively. This behavior is reflected by the smoother post-peak response and the increased deformation capacity observed in the load–displacement curves. The initial stiffness (Ki), calculated as the ratio of Vcr to Dcr, progressively decreased with increasing rubber content, indicating reduced structural rigidity. Likewise, the post-cracking stiffness (Ku) decreased, confirming that rubberized concrete promotes a more deformable structural response. At comparable load levels, the rubberized specimens exhibited larger lateral displacements than the control specimen, indicating greater deformation capacity before reaching the ultimate state.

The ductility index values presented in Table 4 indicate that all specimens exhibited substantial deformation capacity under monotonic loading. Although a slight reduction in the calculated ductility index was observed with increasing rubber content, this trend is mainly associated with the increase in the cracking displacement (Dcr) used in the adopted calculation method. In contrast, the total absorbed energy, calculated as the area under the load–displacement curve, increased steadily with increasing rubber content, reaching approximately 5000 kN·mm for BCJ–CR20. Furthermore, the observed crack patterns and failure modes indicate a transition from a relatively brittle beam flexural failure in the control specimen to a more distributed flexure–shear interaction in the rubberized specimens. Overall, the combined interpretation of Fig. 6; Table 4 demonstrates that incorporating crumb rubber reduces load-carrying capacity and stiffness while significantly improving deformation capacity, energy absorption, and damage tolerance under monotonic loading.

The ductility index reported in Table 4 was calculated as the ratio between the ultimate displacement (Du) and the displacement at first visible cracking (Dcr), as expressed in Eq. (1):

1 $${\upmu}=\frac{{D}_{\mathrm{u}}}{{\mathrm{D}}_{\mathrm{c}\mathrm{r}}}$$

where Du is the ultimate displacement corresponding to the maximum load, and Dcr is the displacement at the first visible cracking. This parameter was used as a comparative indicator of the deformation capacity of the tested specimens under monotonic loading.

The energy absorption capacity of each specimen was evaluated from the experimental load–displacement response. It was calculated as the total area under the load–displacement curve up to the ultimate displacement, representing the amount of mechanical energy absorbed by the specimen before failure. Mathematically, the absorbed energy can be expressed as:

2 $$\mathrm{E}\mathrm{A}=\underset{0}{\overset{{\mathrm{D}}_{\mathrm{u}}}{\int}}\mathrm{P}\left(\mathrm{D}\right)\mathrm{d}\mathrm{D}$$

where EA is the energy absorption capacity (kN·mm), P(D) is the applied load corresponding to the displacement D, and Du is the ultimate displacement. The integration was performed numerically using the experimental load–displacement data for each specimen.

A comparison between the best- and worst-performing specimens further illustrates the influence of crumb rubber incorporation. The control specimen (BCJ–CR0) exhibited the highest ultimate load capacity and initial stiffness because of the superior mechanical properties of conventional concrete. In contrast, specimen BCJ–CR20 showed the lowest strength but achieved the largest ultimate displacement and the highest energy absorption owing to the enhanced deformability provided by the rubber particles. This comparison demonstrates the trade-off between strength and ductility, indicating that moderate rubber incorporation may provide a balanced structural response under monotonic loading.

Overall, the load–displacement results demonstrate that crumb rubber incorporation reduces strength and stiffness but significantly enhances deformation capacity and energy absorption under monotonic loading.

**Table 4:** Experimental response parameters of tested rubberized concrete beam–column joints.

Specimen ID	Vcr (kN)	Dcr (mm)	Vu (kN)	Du (mm)	Ki (kN/mm)	Ku (kN/mm)	Ductility	Total energy (kN·mm)	Failure mode
BCJ–CR0	22.0	1.5	36.5	140	14.7	0.26	93.3	4200	Beam flexural (Relatively Brittle)
BCJ–CR10	20.0	1.8	33.5	160	11.1	0.21	88.9	4500	Flexure–shear interaction (Moderately Ductile)
BCJ–CR15	18.0	2.0	29.0	170	9.0	0.17	85.0	4700	Flexure–shear interaction (Ductile)
BCJ–CR20	15.0	2.2	25.0	190	6.8	0.13	86.4	5000	Flexure–shear interaction (Highly Ductile)

### Crack patterns and failure modes

The failure modes were classified based on visual observations of crack initiation, crack propagation, crack localization, and the dominant failure mechanism observed during testing, following the classification approaches commonly adopted in previous studies on reinforced concrete beam–column joints^13,14^.

**Fig. 7:**
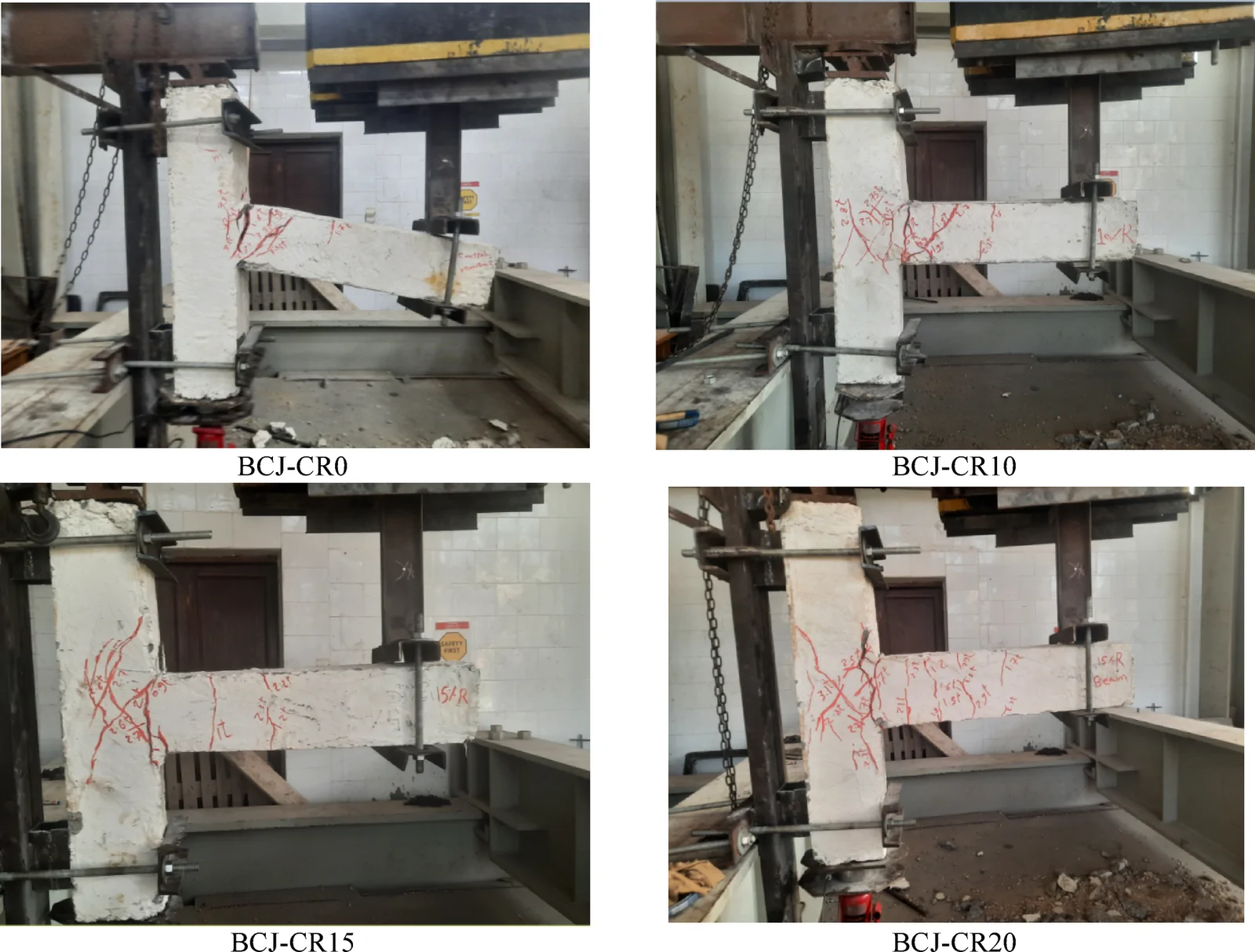
Crack patterns and failure modes of the tested beam-column joint specimens.

The crack patterns and ultimate failure modes of the tested rubberized concrete beam–column joint specimens were carefully monitored throughout the loading process. Representative crack patterns at failure are presented in Fig. 7, while the corresponding structural response parameters and failure classifications are summarized in Table 4. The results clearly indicate that the incorporation of crumb rubber significantly influenced crack development, damage distribution, and the overall failure mechanism.

As shown in Fig. 7, the control specimen (BCJ–CR0) exhibited concentrated cracking near the beam–column interface, with limited crack distribution along the beam. The observed crack pattern indicates a beam flexural-dominated failure characterized by relatively brittle behavior and limited deformation capacity. This specimen also exhibited the highest ultimate load capacity (36.5 kN) and the greatest initial stiffness (14.7 kN/mm), but the lowest energy absorption and deformation tolerance among the tested specimens.

For specimen BCJ–CR10, the crack pattern became more distributed than that of the control specimen. Multiple inclined cracks developed within the joint region and extended into the beam, while crack propagation occurred more gradually. This behavior indicates a reduction in stress concentration and the development of a flexure–shear interaction mechanism. As summarized in Table 4, the specimen exhibited moderate ductility together with an ultimate displacement of 160 mm and higher energy absorption than the control specimen.

The more distributed crack pattern suggests that the rubber particles interrupt the continuity of crack propagation and promote more uniform stress redistribution within the joint region. Consequently, damage evolves progressively rather than through sudden localized cracking, which contributes to the observed enhancement in structural ductility.

Specimen BCJ–CR15 demonstrated a further improvement in crack distribution and deformation behavior. As observed in Fig. 7, cracks became more numerous and spread over a larger portion of both the joint region and the beam, indicating delayed crack localization and more uniform stress redistribution. The structural response was governed by a flexure–shear interaction, accompanied by enhanced ductility, an ultimate displacement of 170 mm, and an absorbed energy of approximately 4700 kN·mm.

The specimen with the highest rubber content (BCJ–CR20) exhibited the most distributed crack pattern and the greatest deformation capacity. Cracks propagated over a wider region of the beam–column connection with less localization around a single dominant crack, demonstrating improved strain accommodation and progressive damage development prior to failure. Although the ultimate load capacity decreased to 25.0 kN, the specimen achieved the highest displacement capacity (190 mm) and the greatest absorbed energy (5000 kN·mm). Accordingly, its structural response was classified as a highly ductile flexure–shear interaction, as presented in Table 4.

Overall, the crack patterns shown in Fig. 7 and the structural response parameters summarized in Table 4 demonstrate that increasing the crumb rubber content gradually changed the governing failure mechanism from a relatively brittle beam flexural response in the control specimen to a more distributed and ductile flexure–shear interaction in the rubberized specimens. Although the incorporation of crumb rubber reduced the ultimate load capacity and stiffness, it significantly enhanced crack distribution, deformation capacity, energy absorption, and damage tolerance under monotonic loading.

Comparing the extreme cases, BCJ–CR0 exhibited localized cracking and relatively brittle failure, whereas BCJ–CR20 developed widely distributed cracks with gradual damage progression. This difference confirms that crumb rubber promotes more uniform stress redistribution and delays crack localization, thereby improving the overall damage tolerance of the beam–column joint.

These observations confirm that increasing the crumb rubber content promotes a more distributed damage pattern and improves the ductile behavior of beam–column joints under monotonic loading.

## Numerical study

A comprehensive three-dimensional nonlinear finite element analysis was conducted using ABAQUS/Standard 2021 (Dassault Systèmes Simulia, Providence, RI, USA) to investigate the structural behavior of rubberized concrete beam–column joints subjected to monotonic loading. The primary objective of the numerical modeling was to accurately reproduce the experimentally observed response in terms of load–displacement behavior, stiffness degradation, crack initiation and propagation, internal stress distribution, and governing failure mechanisms within the joint core.

The concrete material was simulated using the Concrete Damage Plasticity (CDP) model to capture nonlinear phenomena including tensile cracking, compressive crushing, and stiffness degradation. Special attention was given to representing the influence of crumb rubber incorporation on material properties, particularly the reduction in elastic modulus, compressive strength, and bond characteristics.

The developed numerical models were first calibrated and validated against experimental results, including load–displacement curves, crack patterns, and ultimate load capacity, demonstrating good agreement and confirming the reliability of the adopted modeling approach.

Following validation, the numerical model was further utilized to conduct an in-depth parametric study aimed at evaluating the influence of key parameters such as rubber replacement ratio, axial load level, and joint transverse reinforcement on structural performance. In addition, detailed analyses of stress distribution and damage evolution within the joint core were performed to better understand the underlying load transfer mechanisms and the transition in failure modes.

The numerical results provided deeper insight into the role of rubber particles in modifying the internal stress field, reducing stress concentration, and promoting more distributed cracking behavior. This led to a transition from a relatively brittle beam flexural response in the control specimen to a more distributed flexure–shear interaction in the rubberized concrete specimens.

### Finite element modeling

#### Elements and discretization

Three-dimensional nonlinear finite element models were developed using ABAQUS/Standard 2021 (Dassault Systèmes Simulia, Providence, RI, USA) to simulate the behavior of the tested rubberized concrete beam–column joint specimens with high accuracy. The concrete was modeled using eight-node linear brick elements with reduced integration (C3D8R), which are widely used for capturing nonlinear behavior of reinforced concrete, including cracking, stiffness degradation, and damage evolution under complex stress states within joint regions.

A non-uniform meshing strategy was adopted to ensure an optimal balance between computational efficiency and solution accuracy. A refined mesh was applied within the joint core region, as well as near the beam–column interface and loading zones, where high stress gradients and crack localization are expected. In contrast, a relatively coarser mesh was assigned to regions away from the joint to reduce computational cost without compromising overall model performance.

The longitudinal reinforcement in both beam and column, as well as transverse reinforcement within the joint region, were modeled using two-node truss elements (T3D2). These reinforcement elements were embedded within the concrete matrix using the embedded region constraint, assuming perfect bond interaction between steel reinforcement and surrounding concrete. Although bond-slip effects were not explicitly modeled, this assumption provides a reasonable approximation for global response and is consistent with widely adopted practices in nonlinear finite element simulations of reinforced concrete joints.

The material properties of concrete were adjusted to account for the influence of crumb rubber incorporation, particularly the reduction in elastic modulus, compressive strength, and tensile strength, which directly affect stiffness and cracking behavior. This adjustment ensures that the numerical model realistically reflects the mechanical characteristics of rubberized concrete.

To ensure numerical stability and mesh objectivity, a mesh sensitivity analysis was conducted by examining different element sizes ranging from 15 mm to 30 mm within the joint core region. The results indicated that an element size of approximately 25 mm provides an optimal compromise between accuracy and computational efficiency, with variations in predicted ultimate load capacity remaining within an acceptable range of less than 5%. Accordingly, this mesh density was adopted for all subsequent analyses.

The numerical model assumes homogeneous concrete material properties, perfect bond between concrete and reinforcing steel through the embedded-region constraint, and isotropic damage evolution represented by the Concrete Damage Plasticity model. These assumptions are commonly adopted in nonlinear finite element analyses of reinforced concrete structures and have been successfully employed in previous experimental–numerical investigations.

#### Rubberized concrete

The mechanical behavior of rubberized concrete was simulated using the Concrete Damage Plasticity (CDP) model implemented in ABAQUS^12^. This constitutive model is capable of representing key nonlinear phenomena, including tensile cracking, compressive crushing, stiffness degradation, and irreversible damage under complex stress states, which are critical for accurately capturing the response of beam–column joints^35^. The compressive constitutive behavior was defined using the Carreira and Chu model, while the tensile response and damage evolution were implemented through the Concrete Damage Plasticity (CDP) model in ABAQUS. The inclusion of crumb rubber was explicitly considered through the reduction in stiffness and strength parameters, reflecting its influence on the mechanical behavior of the material.

#### Concrete constitutive model

The compressive stress–strain relationship of concrete was defined according to the model proposed by Carreira and Chu^36^, expressed as:

3 $$\frac{{{\upsigma\:}}_{c}}{{f}_{c}^{{\prime\:}}}=\frac{\beta\:\left(\frac{{{\upepsilon\:}}_{c}}{{{\upepsilon\:}}_{o}}\right)}{\beta\:-1+{\left(\frac{{{\upepsilon\:}}_{c}}{{{\upepsilon\:}}_{o}}\right)}^{\beta\:}}$$

where

4 $$\beta=\frac{1}{1-\frac{{f}_{c}^{{\prime}}}{{E}_{c}{{\upepsilon}}_{o}}}$$

where f_c_′ is the concrete compressive strength, E_c_ is the elastic modulus of concrete, ε_c_ is the compressive strain, and ε_0_ is the strain corresponding to the peak compressive stress.

**Fig. 8:**
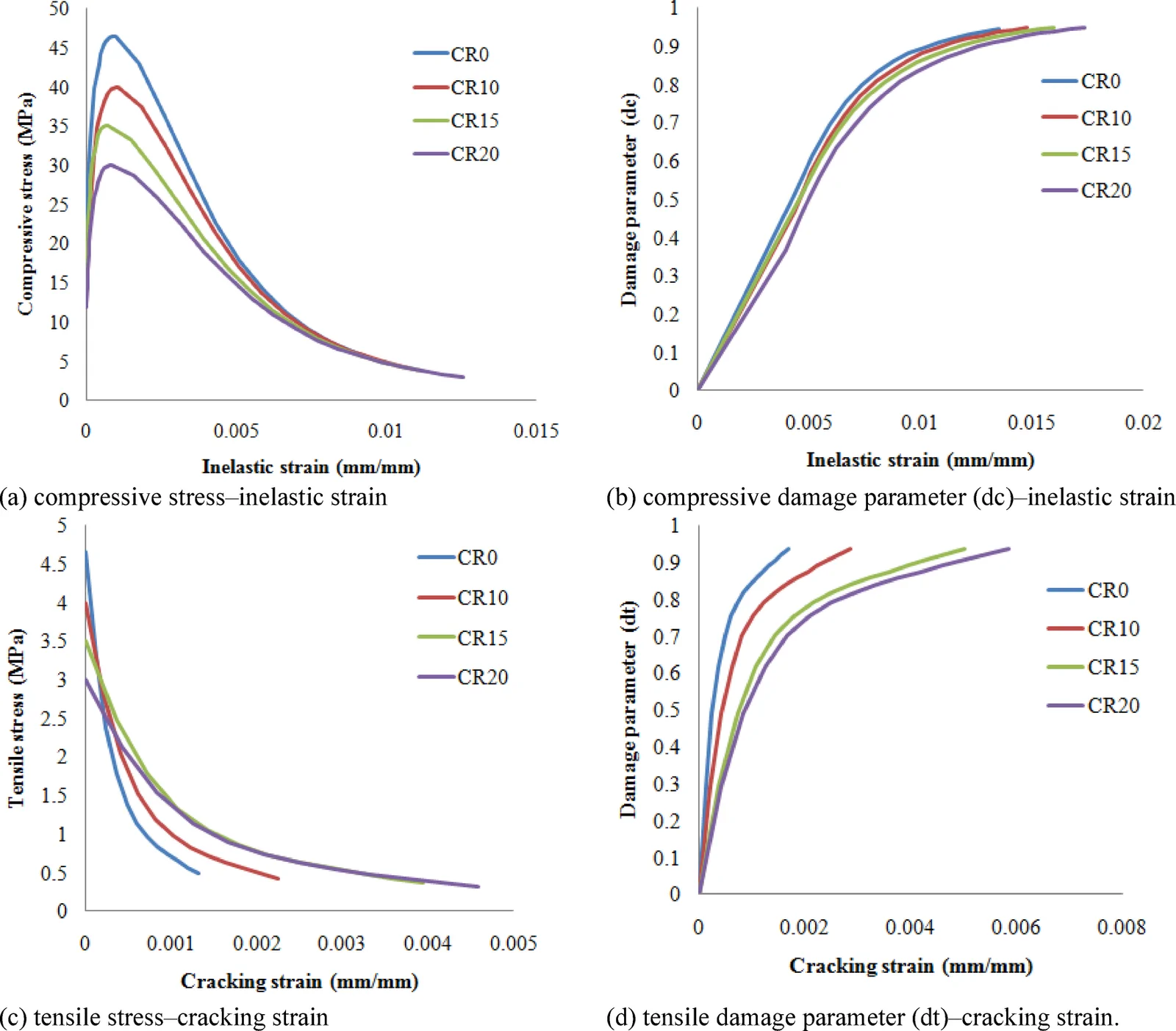
Constitutive relationships adopted in the concrete damage plasticity (CDP) model for CR0, CR10, CR15, and CR20 concrete mixtures.

The tensile constitutive behavior was defined using the tension-softening formulation available in the Concrete Damage Plasticity (CDP) model. The compression damage parameter (dc) and tensile damage parameter (dt) were specified as functions of the corresponding inelastic and cracking strains, respectively. The adopted compression, tension, and damage evolution relationships are presented in Fig. 8.

Each rubberized concrete mixture was modeled as an independent material in ABAQUS by assigning its experimentally measured mechanical properties, including compressive strength, splitting tensile strength, and elastic modulus. The influence of crumb rubber was therefore incorporated indirectly through the experimentally determined material properties rather than by explicitly modeling individual rubber particles. Separate material definitions were created for CR-0, CR-10, CR-15, and CR-20 to accurately represent the influence of crumb rubber content on the constitutive response of concrete.

The tensile response was modeled using a tension-softening law to simulate crack initiation and propagation, along with gradual stiffness degradation after cracking. This approach enables realistic representation of diagonal cracking within the joint core region. The adopted compressive and tensile constitutive relationships, along with the corresponding damage evolution laws, are illustrated in Fig. 8a–d. As shown in Fig. 8a and Fig. 8b, the compressive and tensile stress–strain relationships capture the nonlinear behavior of concrete before and after peak stress. Meanwhile, Fig. 8c and Fig. 8d present the evolution of compressive and tensile damage parameters, respectively, which control stiffness degradation during loading^37^. These damage variables play a key role in simulating crack localization and failure progression within the joint region.

The CDP model parameters, including dilation angle, eccentricity, viscosity parameter, and stress ratios, were selected based on validated values reported in the literature and further calibrated to achieve good agreement with the experimental load–displacement response.

**Fig. 9:**
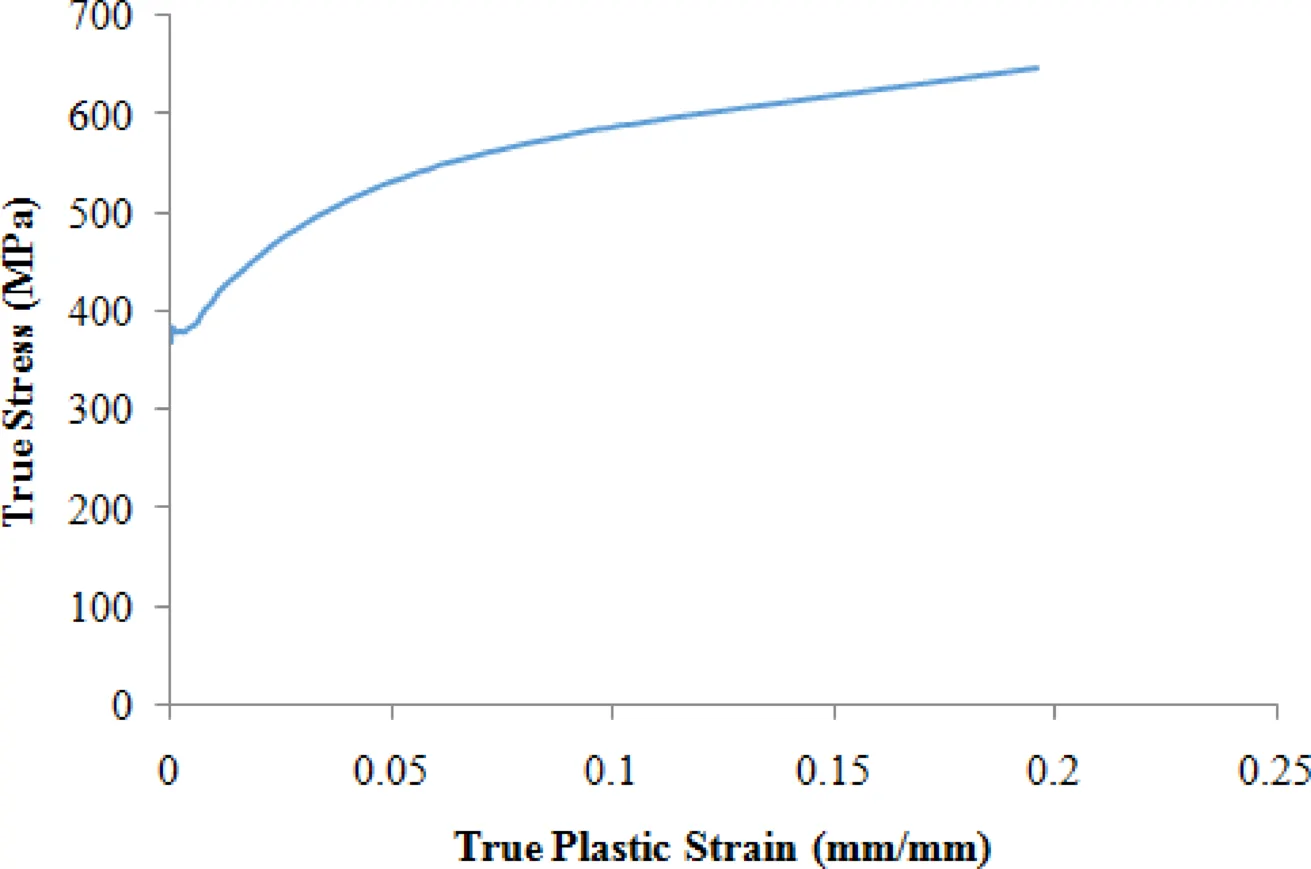
True stress–true plastic strain relationship adopted for reinforcing steel in the finite element model.

The Concrete Damage Plasticity (CDP) model implemented in ABAQUS was adopted to simulate the nonlinear behavior of rubberized concrete. The material model was defined using a Poisson’s ratio of 0.20 for concrete and 0.30 for reinforcing steel, with elastic moduli of 30 GPa and 200 GPa, respectively. The adopted true stress–true plastic strain relationship for reinforcing steel is shown in Fig. 9. The adopted CDP parameters included a dilation angle of 30°, eccentricity of 0.10, fb0/fc0 ratio of 1.16, Kc value of 0.667, and a viscosity parameter of 0.0005. These parameters were selected based on validated values reported in the literature and were further calibrated to achieve good agreement between the numerical simulations and the experimental results.

The Concrete Damage Plasticity (CDP) model was selected because it has been extensively validated for nonlinear analysis of reinforced concrete structures subjected to monotonic and cyclic loading. The adopted CDP parameters were selected based on values commonly reported in previous studies and were verified through calibration against the experimental load–displacement responses of the tested beam–column joint specimens.

This modeling approach allows for a realistic simulation of the transition from initial elastic behavior to nonlinear cracking and eventual failure, enabling accurate prediction of stress redistribution, damage evolution, and failure mechanisms in rubberized concrete beam–column joints.

#### Reinforcement modeling

The reinforcement in both beam and column regions was modeled using an elastoplastic constitutive relationship to accurately capture the nonlinear behavior of steel under monotonic loading. The longitudinal reinforcement bars were represented using a bilinear stress–strain model with isotropic hardening, defined by the experimentally measured yield strength and elastic modulus. An elastic modulus of 200 GPa was assigned to the steel reinforcement, while yielding was defined based on the specified yield stress of the reinforcement bars. Post-yield behavior was incorporated to account for strain hardening, enabling realistic simulation of plastic deformation and energy dissipation under increasing load levels.

The transverse reinforcement (stirrups) within the joint region was modeled using the same constitutive approach, allowing for accurate representation of confinement effects and shear resistance enhancement within the joint core. All reinforcement elements were embedded within the concrete matrix using the embedded region constraint, assuming perfect bond interaction between steel and concrete. Although bond-slip behavior was not explicitly considered, this assumption is widely accepted for simulating the global response of reinforced concrete joints and provides a reasonable approximation for the interaction mechanism. This modeling approach enables accurate prediction of reinforcement yielding, stress redistribution, and confinement effects, which are essential for capturing the nonlinear response and failure mechanisms of rubberized concrete beam–column joints.

The constitutive relationships adopted for both concrete and reinforcing steel were selected to ensure realistic simulation of tensile cracking, compressive crushing, stiffness degradation, and reinforcement yielding throughout the loading history.

#### Steel constitutive model

The reinforcing steel was modeled using an elastic–plastic constitutive relationship. Since ABAQUS requires true stress–plastic strain data as input for metal plasticity, the experimentally measured engineering stress–strain curves were converted into true stress, true strain, and plastic strain using Eqs. (5)–(7), following the standard stress–strain transformation procedures implemented in ABAQUS and commonly adopted in nonlinear finite element analyses of reinforced concrete structures^13^.

5 $${{\upsigma}}_{\mathrm{t}\mathrm{r}\mathrm{u}\mathrm{e}}={{\upsigma}}_{\mathrm{e}\mathrm{n}\mathrm{g}}(1+{{\upepsilon}}_{eng})$$

6 $${{\upepsilon}}_{\mathrm{t}\mathrm{r}\mathrm{u}\mathrm{e}}=\mathrm{L}\mathrm{n}(1+{{\upepsilon}}_{eng})$$

7 $${{\upepsilon}}_{\mathrm{P}\mathrm{l}}={{\upepsilon}}_{\mathrm{t}\mathrm{r}\mathrm{u}\mathrm{e}}-\frac{{{\upsigma}}_{\mathrm{t}\mathrm{r}\mathrm{u}\mathrm{e}}}{{E}_{s}}$$

where σ_eng_ and ε_eng_ are the engineering stress and strain obtained from the tensile tests, σ_true_ and ε_true_ are the corresponding true stress and true strain, ε_pl_ is the plastic strain, and E_s_ is the elastic modulus of steel.

#### Mesh convergence analysis

To ensure that the adopted mesh size provides reliable numerical predictions while maintaining reasonable computational efficiency, a mesh convergence analysis was performed. Five different global mesh sizes (40, 30, 25, 20, and 15 mm) were examined. The predicted ultimate load, total number of elements, and computational time were compared for each mesh. As presented in Table 5, the variation in the predicted ultimate load became insignificant when the mesh size was refined below 25 mm, whereas the computational cost increased considerably. Therefore, a global mesh size of 25 mm was adopted in all subsequent finite element analyses as an optimal balance between numerical accuracy and computational efficiency.

**Table 5:** Mesh convergence analysis used to determine the optimum global mesh size for the finite element model.

Mesh size (mm)	Number of elements	Predicted ultimate load (kN)	Difference (%)	CPU time (min)
40	18,500	35.62	–	8
30	31,700	36.18	1.57	15
25	44,900	36.41	0.64	23
20	69,800	36.47	0.16	38
15	121,500	36.50	0.08	71

Difference (%) represents the percentage variation in the predicted ultimate load relative to the previous mesh size.

#### Boundary conditions and loading

The boundary conditions were defined to reproduce the experimental setup as closely as possible. The base of the column was modeled as a fully restrained support (Encastre) to represent the high rigidity provided by the experimental steel reaction frame. At the top of the column, the axial compressive load was applied through a reference point coupled to the column end, allowing a uniform transfer of the applied load while maintaining compatibility between the loading system and the specimen. The applied axial load corresponded to an axial load ratio of 0.10 and was kept constant throughout the analysis. Monotonic lateral loading was then applied at the free end of the beam under displacement-controlled conditions, consistent with the experimental loading configuration.

Monotonic lateral loading was applied at the free end of the beam in a displacement-controlled manner. This approach ensures numerical stability and enables accurate simulation of nonlinear behavior, including stiffness degradation, crack propagation, and post-peak response up to failure. To prevent local stress concentrations and ensure uniform load transfer, the applied loads were introduced through rigid loading plates. These plates distribute the applied displacement evenly across the loading region, improving the accuracy of the numerical simulation.

**Fig. 10:**
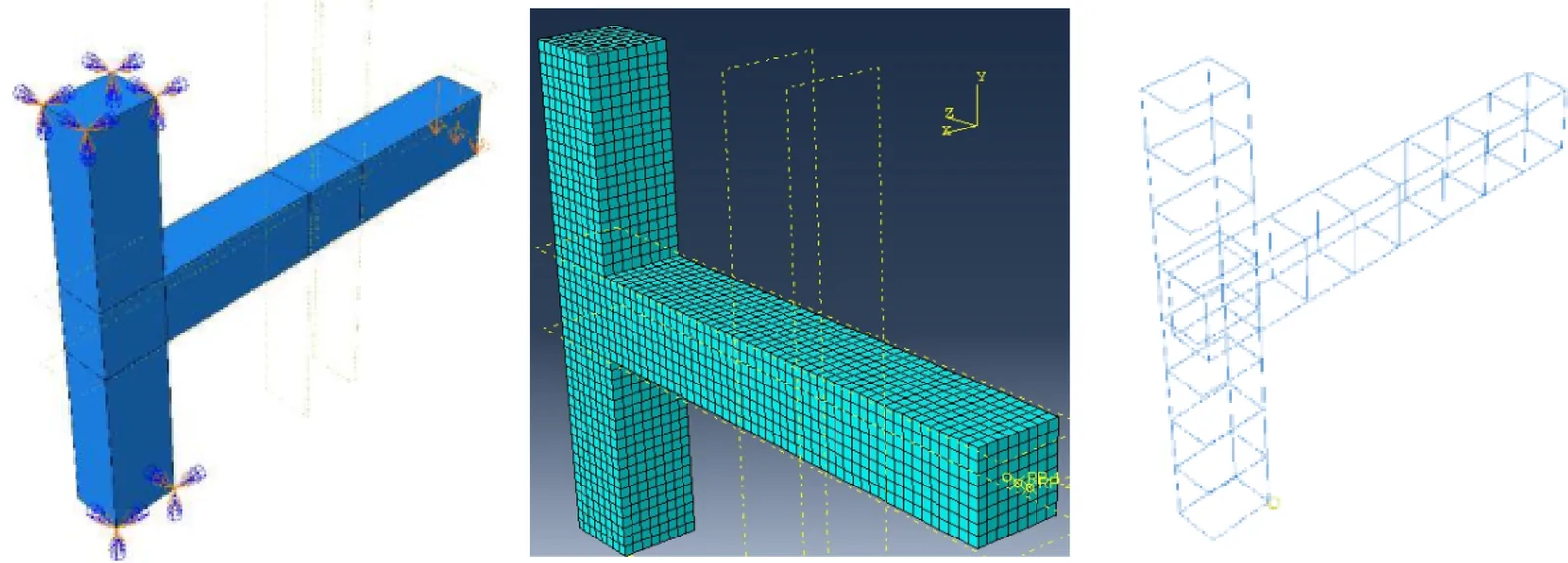
Finite element model configuration of the beam–column joint, including boundary conditions, axial and lateral loading scheme, reinforcement layout, and mesh discretization.

The interaction between boundary conditions and loading configuration plays a critical role in governing stress distribution within the joint core. The adopted setup enables realistic simulation of diagonal strut formation, shear transfer mechanisms, and failure mode development. The overall finite element model configuration, including boundary constraints, loading scheme, reinforcement layout, and mesh discretization, is presented in Fig. 10, where the concrete solid elements, embedded reinforcement, and refined joint core mesh can be clearly observed.

### Finite element model validation

The developed three-dimensional finite element (FE) models were validated against the experimental results obtained from the tested rubberized concrete beam–column joint specimens. The validation was performed through a comprehensive comparison of the load–displacement responses, ultimate load capacities, and overall structural behavior.

**Fig. 11:**
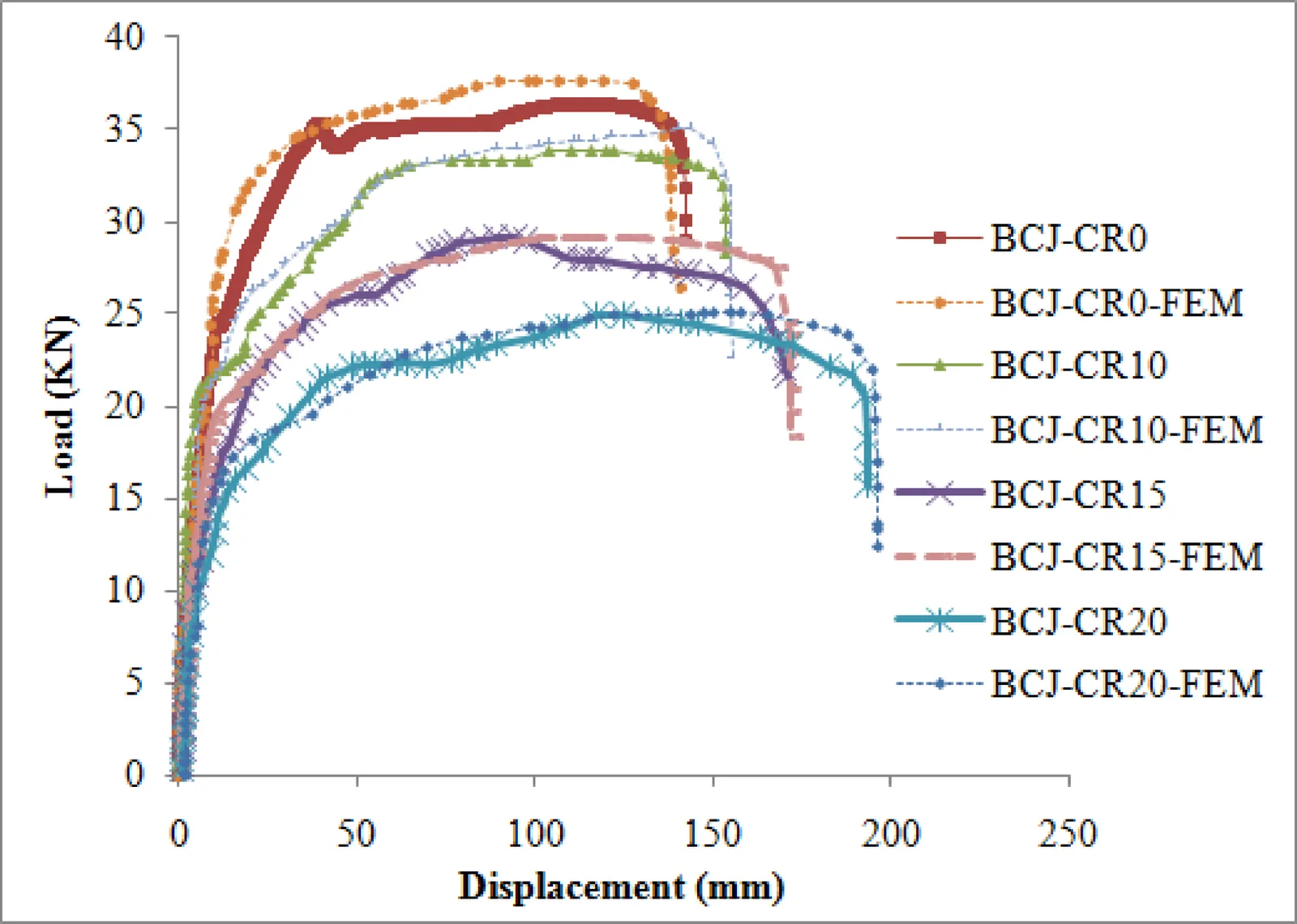
Comparison between experimental and finite element (FE) load–displacement curves for rubberized concrete beam–column joint specimens.

The representative experimental and numerical load–displacement curves are presented in Fig. 11, where good agreement between both responses can be observed for all specimens. As shown in Fig. 11, the FE simulations successfully capture the initial stiffness, crack initiation, nonlinear response, and ultimate load of the tested beam–column joints. The numerical curves closely follow the experimental responses throughout the ascending branch and near the peak load, demonstrating the capability of the developed model to reproduce the overall structural behavior.

For all specimens, the numerical model successfully reproduces the influence of crumb rubber incorporation on structural performance. The control specimen (BCJ–CR0) exhibited the highest stiffness and load-carrying capacity, whereas the rubberized specimens (BCJ–CR10, BCJ–CR15, and BCJ–CR20) showed progressively lower stiffness and strength together with enhanced deformation capacity as the rubber replacement ratio increased. These response trends were consistently captured by the FE simulations, confirming the ability of the numerical model to realistically represent the effect of crumb rubber on the structural behavior of beam–column joints.

A quantitative comparison between the experimental and numerical results is summarized in Table 6. The predicted ultimate load capacities are in very good agreement with the experimental measurements, with deviations ranging from only 1.03% to 5.07%. Likewise, the predicted ultimate displacements show reasonable agreement with the experimental results. Although the displacement errors are relatively larger than those associated with the ultimate loads, they remain acceptable considering the highly nonlinear post-cracking behavior of reinforced concrete beam–column joints. The relatively larger discrepancies in ultimate displacement are mainly attributed to the simplified representation of post-cracking stiffness degradation, bond-slip behavior, and damage localization in the numerical model, whereas the prediction of ultimate load remained highly accurate.

The small discrepancies between the experimental and numerical responses can be attributed to several modeling assumptions adopted in the finite element analysis. First, the embedded region constraint assumes perfect bond between the reinforcing steel and the surrounding concrete, whereas bond degradation and local reinforcement slip may occur during the experimental tests. Second, the Concrete Damage Plasticity (CDP) model represents concrete deterioration using a smeared-crack approach, which cannot fully reproduce localized crack propagation and fracture development observed experimentally. Furthermore, the numerical model assumes homogeneous material properties and ideal geometric conditions, while the experimental specimens inevitably contain material heterogeneity, construction tolerances, and minor geometric imperfections. In addition, residual stresses, local microcracking prior to loading, and uncertainties associated with concrete casting, curing conditions, and the distribution of crumb rubber particles are not explicitly represented in the numerical model. These unavoidable simplifications explain the small differences observed between the experimental and numerical responses.

Despite these minor deviations, the FE model successfully reproduces both the global structural response and the relative performance trends among the tested specimens. The close agreement between the experimental and numerical results, as demonstrated in Fig. 11; Table 6, confirms the reliability of the adopted modeling approach and demonstrates that the Concrete Damage Plasticity (CDP) model adequately captures the dominant nonlinear mechanisms governing rubberized concrete beam–column joints, including stiffness degradation, tensile cracking, compressive damage, and progressive failure under monotonic loading. Therefore, the validated numerical model provides a reliable analytical framework for conducting further parametric investigations and for gaining deeper insight into the stress distribution, damage evolution, and structural behavior of rubberized concrete beam–column joints beyond the limitations of the experimental program.

**Table 6:** Comparison between experimental and numerical (FE) results of rubberized concrete beam–column joint specimens.

Beam ID	Vu, exp (kN)	Vu, FE (kN)	Error in Vu (%)	Δu, exp (mm)	Δu, FE (mm)	Error in Δu (%)
BCJ–CR0	36.5	37.6	**3.01%**	140	141.26	0.90%
BCJ–CR10	33.5	35.2	5.07%	160	144.8	-9.50%
BCJ–CR15	29.0	29.3	1.03%	170	149.8	-11.88%
BCJ–CR20	25.0	25.9	3.60%	190	196	3.16%

#### Crack patterns and failure modes

**Fig. 12:**
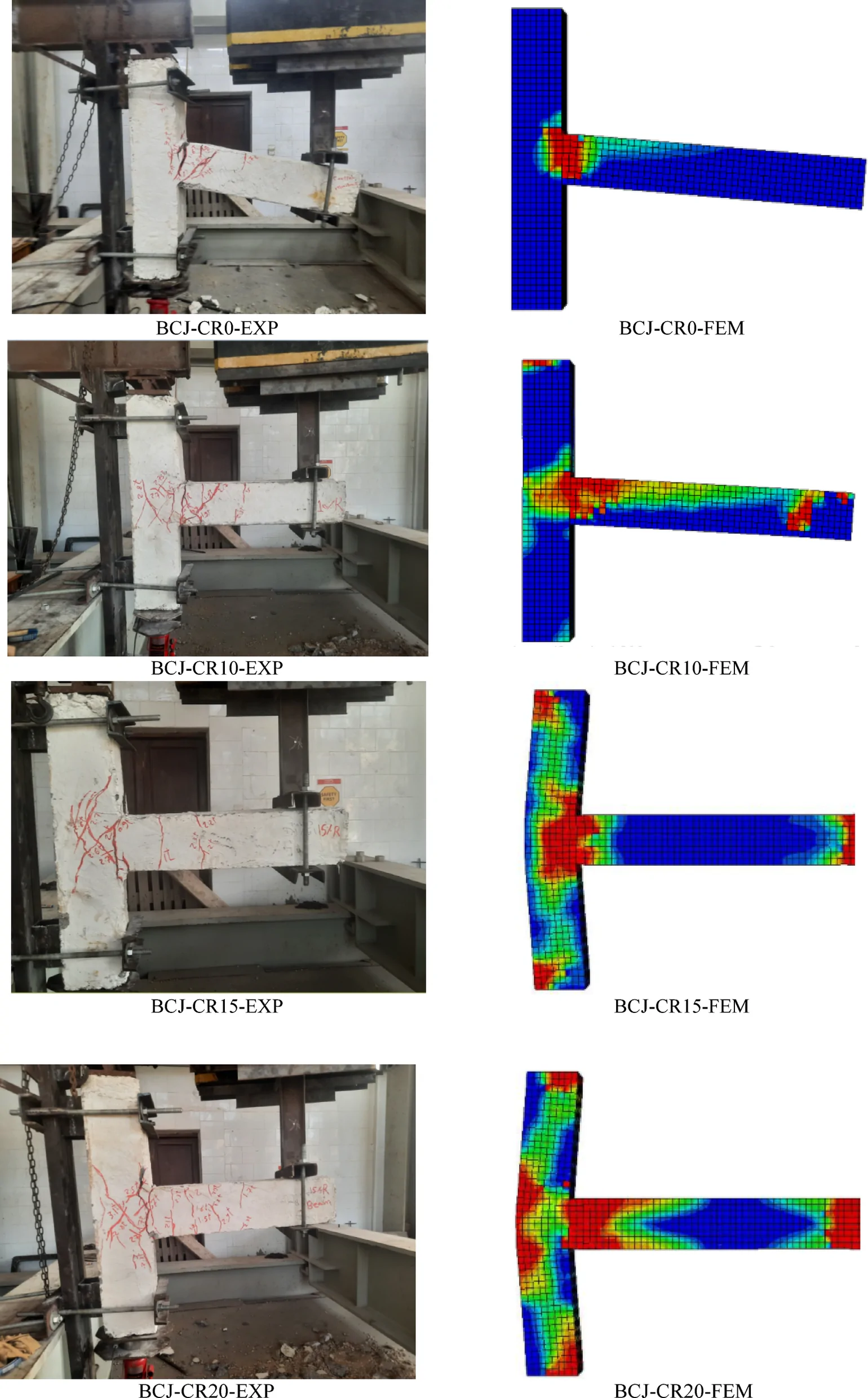
Comparison between experimentally observed crack patterns and tensile damage contours (DAMAGET).

The crack patterns and failure modes of the tested rubberized concrete beam–column joint specimens were evaluated through a direct comparison between the experimentally observed crack maps and the tensile damage contours obtained from the finite element (FE) simulations, as illustrated in Fig. 12. The numerical models demonstrated a strong capability in accurately reproducing the crack initiation, propagation paths, and damage distribution observed experimentally within both the beam and the joint region.

For the control specimen (BCJ–CR0), the experimental observations indicated a beam flexural-dominated failure, characterized by concentrated flexural cracking near the beam–column interface and relatively limited crack distribution along the beam. The FE model accurately reproduced this behavior by predicting localized tensile damage concentrated in the beam adjacent to the joint region, together with limited damage within the joint core. The close agreement between the experimental crack pattern and the numerical damage contours confirms the capability of the adopted FE model to accurately capture the governing failure mechanism of the control specimen.

With the incorporation of crumb rubber (BCJ–CR10 and BCJ–CR15), the crack patterns became more distributed, with multiple inclined and flexural cracks propagating along the beam and extending into the joint region. Experimentally, crack propagation occurred more gradually and damage was distributed over a wider area compared with the control specimen. The FE simulations successfully reproduced this behavior, showing wider tensile damage zones and a more uniform strain distribution, indicating reduced stress concentration and enhanced energy dissipation capacity. These observations confirm that the governing structural response gradually shifted from localized beam flexural damage toward a flexure–shear interaction.

For the specimen containing the highest rubber content (BCJ–CR20), the structural response exhibited the greatest deformation capacity together with the most distributed cracking pattern. Cracks propagated over a larger portion of the beam and joint region with reduced localization, indicating progressive damage development prior to failure. The FE damage contours closely reflected this response by predicting a broader distribution of tensile damage rather than localization along a single dominant crack path. This behavior highlights the beneficial role of crumb rubber in modifying the internal stress field, improving stress redistribution, and delaying crack localization.

In addition to the tensile damage distribution, the FE simulations indicated that compressive stress concentrations remained primarily located near the beam–column interface and within the joint region. However, the intensity of these stress concentrations gradually decreased with increasing crumb rubber content, reflecting a more uniform stress transfer mechanism and improved damage tolerance.

Overall, the excellent agreement between the experimentally observed crack patterns and the tensile damage (DAMAGET) contours predicted by the FE model confirms that the adopted numerical approach accurately captures the evolution of cracking, damage propagation, and the governing failure mechanisms of rubberized concrete beam–column joints under monotonic loading. Both the experimental observations and the numerical results demonstrate that increasing crumb rubber content promotes more uniform stress redistribution, delays crack localization, and changes the structural response from a relatively brittle beam flexural-dominated failure in the control specimen to a more distributed and ductile flexure–shear interaction in the rubberized specimens.

#### Model reliability and limitations

The strong agreement observed between the experimental results and the finite element (FE) predictions in terms of load–displacement response, ultimate load capacity, crack patterns, and failure modes confirms the reliability of the developed numerical models for simulating the behavior of rubberized concrete beam–column joints under monotonic loading. The validated models successfully reproduced the initial stiffness, crack initiation and propagation, nonlinear response up to failure, as well as the governing structural response observed experimentally.

Furthermore, the FE simulations accurately captured the influence of the key governing parameters, including crumb rubber replacement ratio, column axial load level, and joint confinement provided by transverse reinforcement. In particular, the numerical model successfully reproduced the gradual transition from the relatively brittle beam flexural-dominated failure observed in the control specimen (BCJ–CR0) to the more distributed and ductile flexure–shear interaction exhibited by the rubberized specimens (BCJ–CR10, BCJ–CR15, and BCJ–CR20). This close agreement between the experimental observations and the numerical damage contours further demonstrates the robustness and predictive capability of the adopted modeling approach.

Despite the overall accuracy of the numerical simulations, certain modeling assumptions introduce inherent limitations. The use of the embedded-region constraint assumes perfect bond between reinforcing steel and the surrounding concrete, which may lead to a slight overestimation of structural stiffness, particularly during the post-cracking stage where bond deterioration and slip effects become more significant. In addition, the incorporation of crumb rubber modifies the interfacial transition zone and introduces localized deformation mechanisms that cannot be fully represented by the adopted constitutive model.

Moreover, the simplified representation of shear-transfer mechanisms, including aggregate interlock, reinforcement dowel action, and crack-surface roughness, may influence the accuracy of the predicted post-peak response and crack-width evolution. Although the Concrete Damage Plasticity (CDP) model effectively captures the global nonlinear response and damage evolution, it adopts a smeared-crack formulation that cannot fully reproduce localized cracking and concrete crushing observed experimentally.

Nevertheless, considering the relatively small deviations between the experimental and numerical predictions of ultimate load, displacement capacity, crack patterns, and failure modes, the developed FE models can be considered both robust and reliable. Therefore, the validated numerical framework provides a sound basis for conducting advanced parametric investigations and developing performance-based design recommendations for rubberized concrete beam–column joints.

The observed differences between the experimental and numerical responses fall within the range commonly reported for nonlinear finite element simulations of reinforced concrete structures and are therefore considered acceptable.

It should be noted that the investigated specimens were governed by beam flexural-dominated failure or flexure–shear interaction, rather than pure joint shear failure. Consequently, direct comparison with existing joint shear design provisions was beyond the scope of the present study. Future investigations employing specimens intentionally designed to develop joint shear failure are recommended to evaluate the applicability of current design-code provisions to rubberized concrete beam–column joints.

### Parametric study

The objective of the parametric study was not only to confirm the expected behavioral trends but also to quantify the relative influence of key design parameters on the structural performance of rubberized concrete beam–column joints after validating the numerical model against the experimental results.

Following the successful validation of the finite element model, a comprehensive parametric study was carried out using specimen BCJ–CR15 as the reference model because it exhibited a balanced response in terms of strength, ductility, and damage tolerance. The study was intended to examine the influence of the main structural variables on the monotonic behavior of rubberized concrete beam–column joints while keeping all other parameters constant.

The investigated variables are presented in Table 7 and include the column axial load ratio, concrete compressive strength, beam longitudinal reinforcement ratio, and joint transverse reinforcement ratio. These parameters were selected because of their significant role in controlling joint confinement, load transfer mechanisms, stiffness characteristics, crack propagation, and overall structural capacity.

The influence of these variables was evaluated through load–displacement response, ultimate load capacity, stiffness degradation, ductility, and governing failure mode. The obtained results provide a deeper understanding of the nonlinear behavior of rubberized concrete beam–column joints and establish a rational basis for future performance-based design recommendations.

**Table 7:** Exact parameter values adopted in the parametric study.

Parameter varied	Symbol	Adopted values
Column axial load ratio	n	0.05, 0.10, 0.30, 0.50
Concrete compressive strength (MPa)	f_cu_	30.00, 36.40, 45
Beam longitudinal reinforcement ratio	ρ_b_	0.012, 0.015, 0.018
Joint transverse reinforcement ratio	ρ_j_	0.0025, 0.0035, 0.005

#### Effect of column axial load ratio

**Fig. 13:**
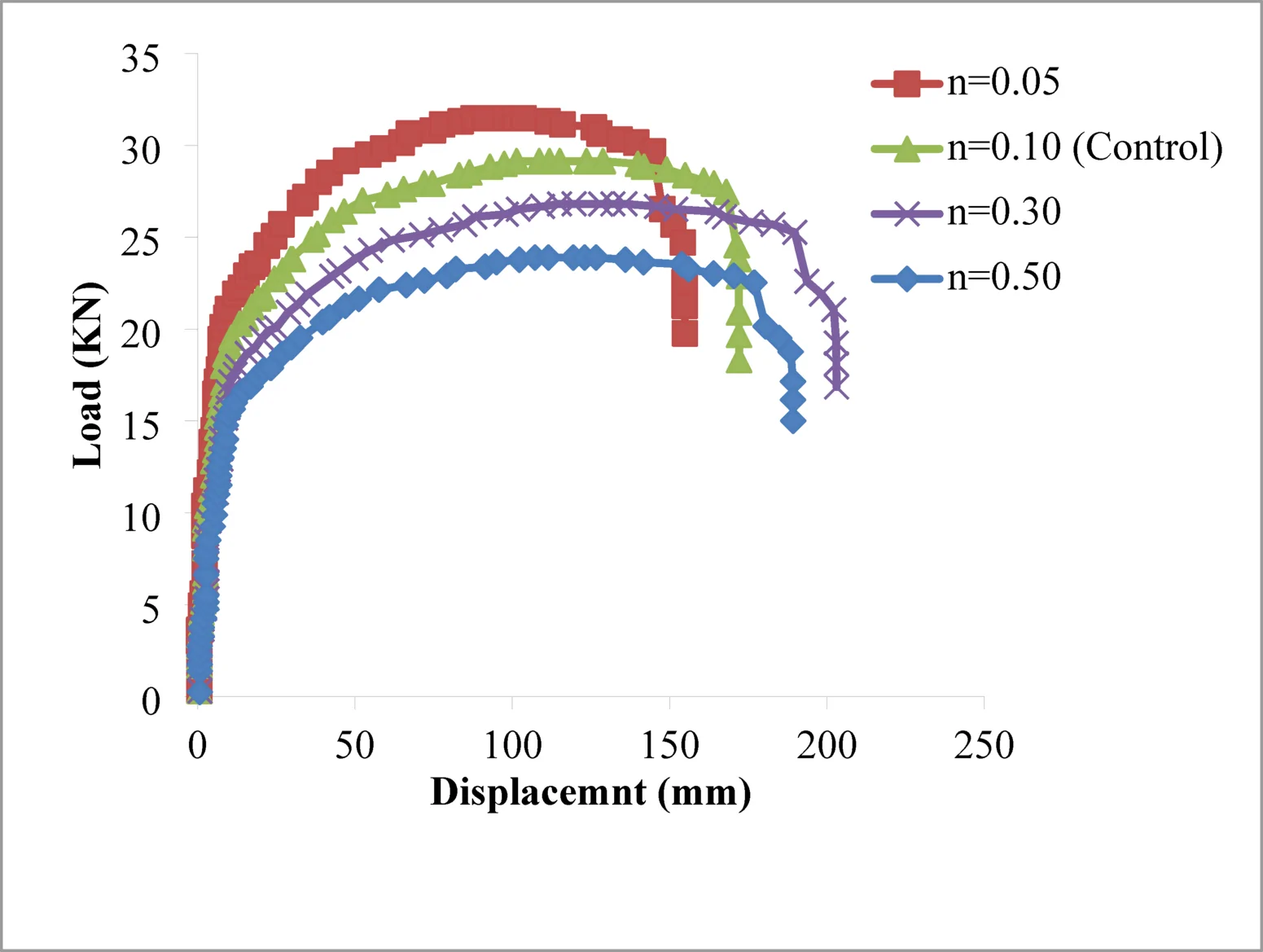
Effect of column axial load ratio.

The influence of the column axial load ratio (n) on the structural response of rubberized concrete beam–column joints was numerically investigated while maintaining all remaining material properties, reinforcement ratios, geometric dimensions, and boundary conditions constant. The corresponding load–displacement responses for different axial load ratios are illustrated in Fig. 13.

As shown in Fig. 13, the column axial load ratio significantly affects the load-carrying capacity, stiffness characteristics, and deformation behavior of the joint specimens. The control model (*n* = 0.10) exhibited a balanced response with moderate ultimate load capacity and stable post-peak behavior, and was therefore adopted as the reference configuration for comparison.

Reducing the axial load ratio to *n* = 0.05 resulted in the highest ultimate load capacity among all investigated cases. The corresponding curve shows a steeper ascending branch and a higher peak load, indicating enhanced stiffness and improved lateral resistance. This behavior may be attributed to the reduced compressive stress level in the column, which delays concrete crushing within the joint core and allows more efficient stress redistribution during loading.

Increasing the axial load ratio beyond the control level to *n* = 0.30 caused a moderate reduction in ultimate load capacity, although the specimen maintained relatively stable post-peak behavior and exhibited the largest displacement capacity. The extended descending branch indicates improved deformation tolerance and gradual strength degradation. This suggests that moderate axial compression can still provide beneficial confinement while allowing sustained inelastic response.

Further increasing the axial load ratio to *n* = 0.50 resulted in the lowest ultimate load capacity and the softest structural response. The load–displacement curve exhibits reduced peak strength and earlier post-peak degradation, indicating premature crushing and higher stress concentration within the joint region. Excessive axial compression tends to accelerate concrete damage, reduce lateral deformation capacity, and limit the ability of the joint to sustain increasing load.

Overall, the results presented in Fig. 13 confirm that the column axial load ratio is a key governing parameter influencing the monotonic behavior of rubberized concrete beam–column joints. Lower-to-moderate axial load ratios provide favorable structural performance, whereas excessive axial compression leads to strength deterioration and reduced ductility. Within the investigated range, the most efficient response was observed at *n* = 0.05, while the control case (*n* = 0.10) maintained a desirable balance between strength and deformation capacity.

#### Effect of concrete compressive strength

**Fig. 14:**
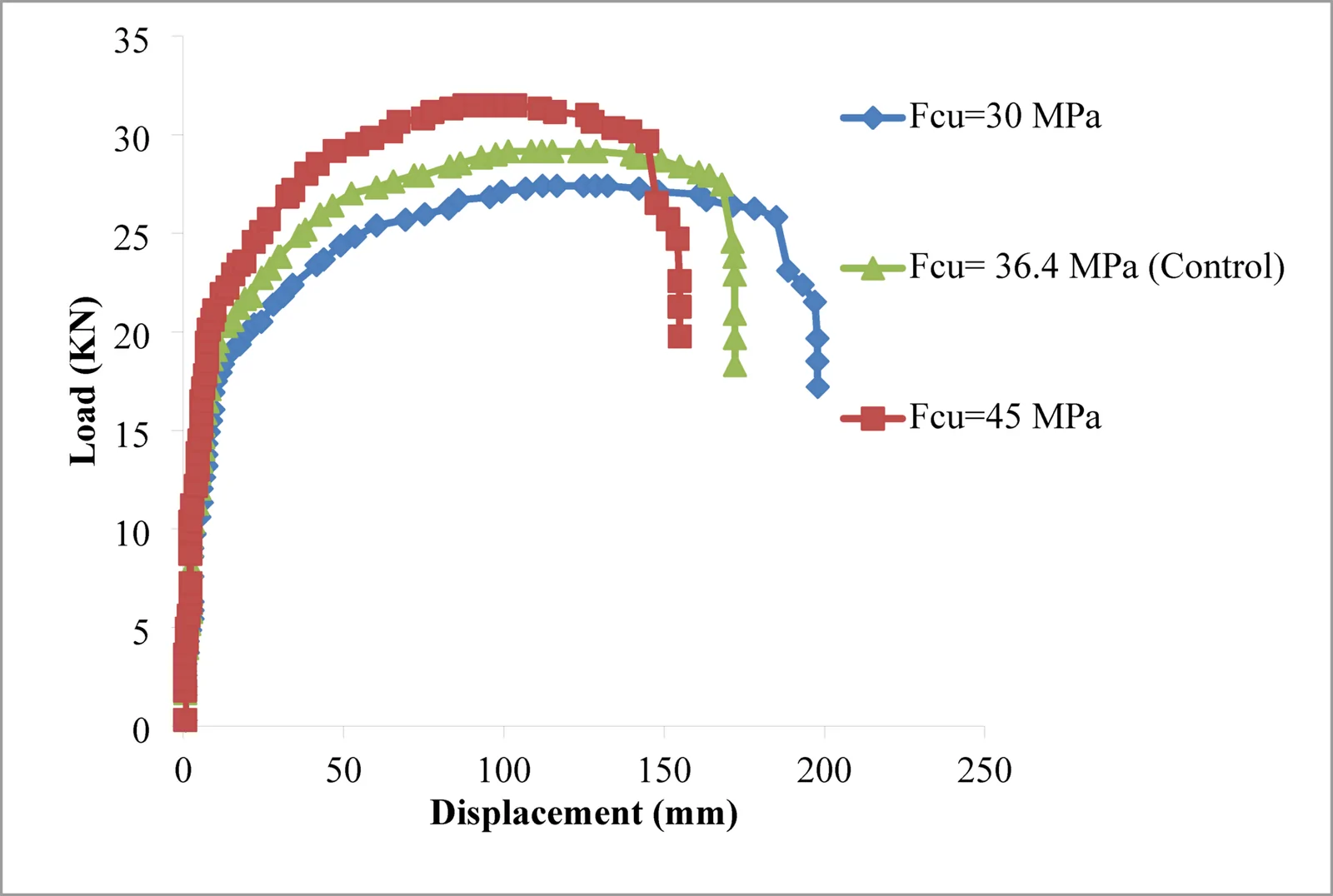
Effect of concrete compressive strength.

The influence of concrete compressive strength (Fcu) on the monotonic response of rubberized concrete beam–column joints was investigated numerically, and the corresponding load–displacement curves are presented in Fig. 14. As shown in Fig. 14, increasing Fcu from 30 MPa to 45 MPa enhanced both the initial stiffness and peak load capacity of the joint specimens. The model with Fcu = 45 MPa exhibited the highest ultimate load (approximately 31.5 kN), followed by the control model (36.4 MPa) and the 30 MPa model. This behavior is attributed to the improved compressive resistance of the joint core and more efficient internal force transfer at higher concrete strengths.

However, the specimen with Fcu = 30 MPa developed the largest displacement capacity and the most gradual post-peak response, indicating superior deformation tolerance. In contrast, the 45 MPa model showed a sharper descending branch after peak load, reflecting the relatively more brittle behavior commonly associated with higher-strength concrete. The control specimen (Fcu = 36.4 MPa) provided a balanced structural response by combining moderate strength, stable post-peak behavior, and adequate deformation capacity.

Overall, the results in Fig. 14 indicate that concrete compressive strength governs the trade-off between strength and ductility. Higher strengths improve load capacity and stiffness, whereas lower strengths enhance displacement capacity. Therefore, an intermediate concrete strength appears more suitable for achieving balanced performance in rubberized concrete beam–column joints.

#### Effect of beam longitudinal reinforcement ratio

**Fig. 15:**
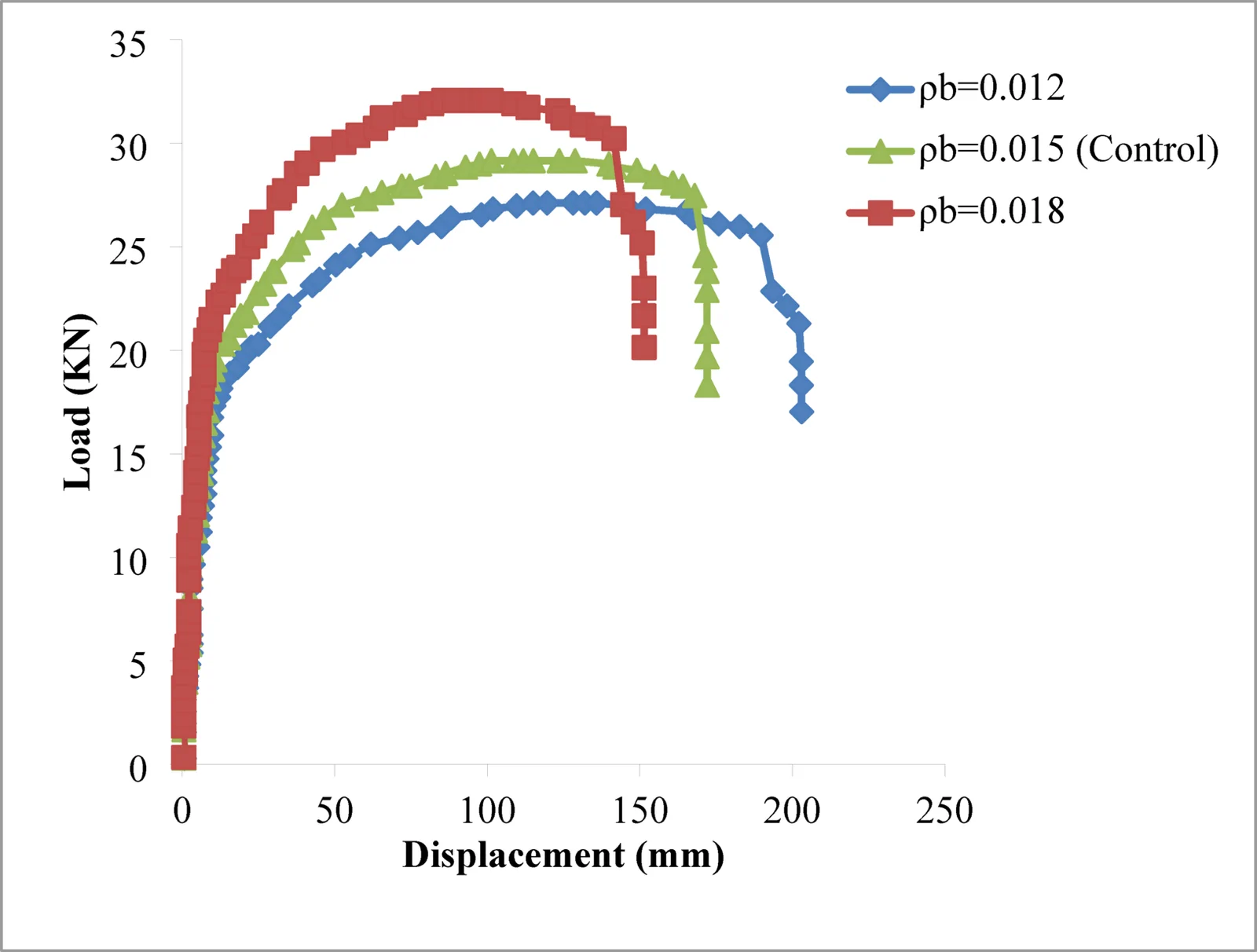
Effect of Beam longitudinal reinforcement ratio.

The influence of the beam longitudinal reinforcement ratio (ρb) on the monotonic response of rubberized concrete beam–column joints was numerically investigated, and the corresponding load–displacement curves are presented in Fig. 15. As shown in Fig. 15, increasing ρb from 0.012 to 0.018 improved the initial stiffness and ultimate load capacity of the joint specimens. The model with ρb = 0.018 exhibited the highest peak load (approximately 32 kN), followed by the control model (ρb = 0.015) and the ρb = 0.012 model. This trend reflects the contribution of additional longitudinal reinforcement to beam flexural resistance and more efficient force transfer to the joint region.

However, the specimen with ρb = 0.012 developed the largest displacement capacity, reaching approximately 210 mm, together with a more gradual post-peak response. In contrast, the ρb = 0.018 model showed earlier strength degradation after peak load, indicating reduced deformation capacity due to the increased stiffness of the reinforced beam.

The control specimen (ρb = 0.015) provided a balanced response, combining moderate strength, stable post-peak behavior, and adequate displacement capacity. Overall, the results in Fig. 15 indicate that increasing the beam longitudinal reinforcement ratio primarily enhances strength and stiffness, whereas lower reinforcement ratios improve displacement capacity and ductility. Therefore, an intermediate reinforcement ratio appears more suitable for achieving balanced structural performance in rubberized concrete beam–column joints.

#### Effect of joint transverse reinforcement ratio

**Fig. 16:**
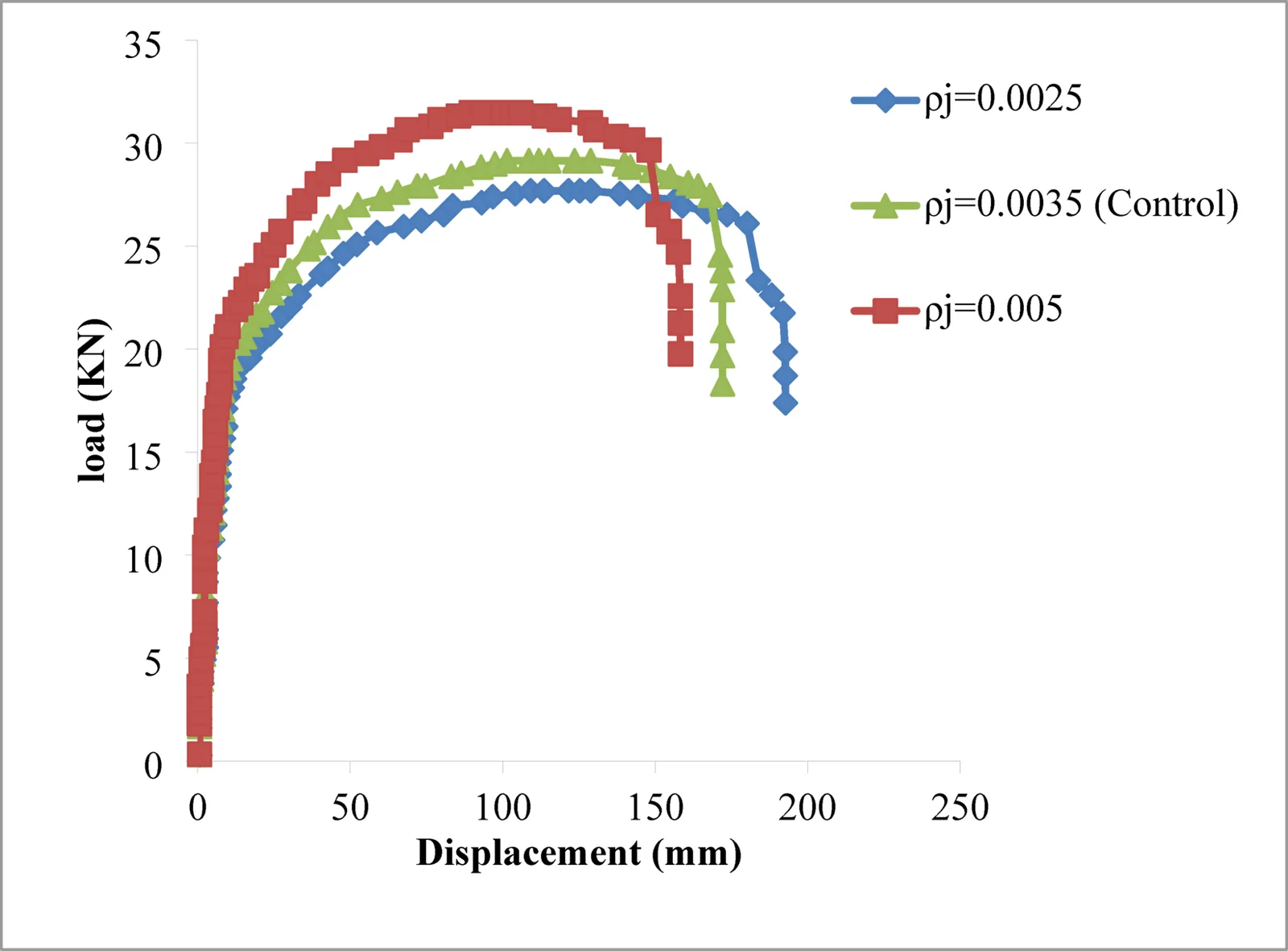
Effect of joint transverse reinforcement ratio.

The influence of the joint transverse reinforcement ratio (ρj) on the monotonic response of rubberized concrete beam–column joints was numerically investigated, and the corresponding load–displacement curves are presented in Fig. 16. As shown in Fig. 16, increasing ρj from 0.0025 to 0.005 enhanced the initial stiffness and ultimate load capacity of the joint specimens. The model with ρj = 0.005 exhibited the highest peak load (approximately 32 kN), followed by the control model (ρj = 0.0035) and the ρj = 0.0025 model. This trend confirms the beneficial role of transverse reinforcement in improving confinement of the joint core and resisting diagonal shear stresses.

The specimen with ρj = 0.0025 developed the largest displacement capacity, reaching approximately 190 mm, but showed a steeper post-peak strength reduction, indicating weaker confinement and faster damage progression after peak load. In contrast, the ρj = 0.005 model exhibited a more stable response before failure, although with lower ultimate displacement. The control specimen (ρj = 0.0035) provided a balanced structural response by combining moderate strength, stable post-peak behavior, and adequate deformation capacity.

Overall, the results in Fig. 16 indicate that increasing the joint transverse reinforcement ratio primarily improves strength, stiffness, and structural stability, whereas lower reinforcement ratios enhance displacement capacity at the expense of confinement efficiency. Therefore, an intermediate-to-high transverse reinforcement ratio is preferable for achieving balanced performance in rubberized concrete beam–column joints.

The conducted parametric study demonstrates that the structural performance of rubberized concrete beam–column joints is governed by the combined interaction of the axial load ratio, concrete compressive strength, and reinforcement detailing rather than by any individual parameter alone. While the observed trends are generally consistent with established structural mechanics principles, the validated finite element model provides quantitative insight into the relative contribution of each design parameter to the load-carrying capacity, stiffness, ductility, and post-peak response of rubberized concrete joints. These findings provide practical guidance for selecting appropriate design parameters and support the structural design and assessment of sustainable rubberized concrete beam–column joints under monotonic loading.

It should be noted that the present parametric investigation is limited to monotonic loading conditions. Therefore, future studies should extend the numerical investigation to cyclic and seismic loading, as well as different joint configurations and wider ranges of material properties, in order to establish more comprehensive design recommendations for rubberized concrete beam–column joints.

## Discussion of results

The present study provides a comprehensive understanding of the monotonic behavior of rubberized concrete beam–column joints through the integration of experimental testing, validated nonlinear finite element modeling, and parametric analysis. The results demonstrate that joint performance is governed by the interaction between material deformability, stiffness characteristics, confinement efficiency, reinforcement detailing, and internal stress redistribution. Unlike conventional concrete joints, where failure is commonly associated with brittle diagonal shear cracking, the incorporation of crumb rubber significantly modified the structural response by reducing stress concentration, delaying crack localization, and promoting a more gradual failure mechanism^38–40^.

The experimental observations obtained in the present study are generally consistent with previous investigations on rubberized concrete structural members. Several researchers have reported that replacing natural aggregates with crumb rubber leads to a reduction in compressive strength and structural stiffness because of the lower elastic modulus of rubber particles and the weaker interfacial transition zone. At the same time, improvements in deformation capacity, crack distribution, and energy absorption have been consistently observed owing to the enhanced flexibility of the rubberized concrete matrix. The present experimental results confirm these trends at the beam–column joint level, while also demonstrating that the incorporation of crumb rubber modifies the governing failure mechanism by promoting a more distributed flexure–shear interaction under monotonic loading.

A major outcome of the experimental investigation is that increasing the rubber replacement ratio from 0% to 20% caused a progressive reduction in ultimate load capacity and initial stiffness. This behavior is mainly attributed to the lower elastic modulus of rubber particles and the weaker interfacial transition zone between rubber and cement paste, which reduce the ability of the concrete matrix to resist compression and shear stresses^41–43^. However, this reduction in strength was accompanied by clear improvements in deformation capacity and absorbed energy. The increase in ductility indicates that rubberized concrete behaves as a more deformable composite capable of sustaining larger inelastic strains before failure. From a structural perspective, this characteristic is beneficial for beam–column joints, where deformation tolerance is often more critical than peak resistance under extreme loading conditions^44,45^.

The observed crack patterns further support this interpretation. In the control specimen, damage remained relatively localized near the beam–column interface, whereas the rubberized specimens exhibited more distributed cracking throughout the joint region and adjacent beam. Crack propagation became more gradual, crack widths were generally smaller, and damage localization was delayed. This behavior suggests that crumb rubber particles interrupted crack continuity, increased fracture energy, and enhanced stress redistribution within the joint, which is consistent with the smoother descending branches observed in the load–displacement responses^46,47^.

The observed transition from a relatively brittle structural response to a more ductile behavior is also in agreement with previous studies, which reported that crumb rubber particles reduce stress concentration and increase fracture energy, thereby delaying crack localization and promoting progressive failure. However, unlike many previous investigations that focused primarily on material properties or flexural members, the present study experimentally demonstrates these effects in reinforced concrete beam–column joints and further validates the observed behavior through nonlinear finite element analysis.

The numerical parametric study confirmed that the column axial load ratio is one of the most influential parameters governing joint behavior. Moderate axial compression improved confinement of the joint core and enhanced diagonal compression strut action, resulting in higher stiffness and more efficient stress transfer. However, excessive axial load caused premature concrete crushing, reduced lateral deformation capacity, and accelerated post-peak strength degradation. This reflects the dual role of axial compression in joint mechanics, where moderate compression is beneficial, while excessive compression reduces the deformation reserve required for nonlinear response^48^. Therefore, axial load should be carefully controlled in practical design.

Concrete compressive strength also showed a significant influence on structural response. Increasing concrete strength enhanced peak load capacity and initial stiffness owing to the higher compressive resistance of the joint core. However, lower concrete strength provided larger displacement capacity and a more gradual post-peak softening response due to reduced brittleness. This indicates that concrete strength controls the balance between load resistance and deformability, and that intermediate strength levels may provide more stable overall behavior.

The influence of the beam longitudinal reinforcement ratio highlights the importance of balanced stiffness design. Increasing beam reinforcement improved ultimate load capacity and initial stiffness due to the higher flexural resistance of the beam and more efficient force transfer to the joint region. However, the higher reinforcement ratios showed lower displacement capacity and faster post-peak degradation, reflecting the effect of increased member stiffness and reduced deformation tolerance. Conversely, lower reinforcement ratios enhanced displacement capacity and post-peak ductility, although with reduced peak strength.

The joint transverse reinforcement ratio had a clear positive effect on strength and structural stability. Increasing transverse reinforcement improved confinement of the joint core, restrained diagonal crack widening, and enhanced resistance to joint shear stresses. The specimens with higher transverse reinforcement ratios developed higher peak loads and more stable pre-peak behavior, while lower ratios exhibited faster post-peak degradation due to insufficient confinement. These findings confirm the importance of joint stirrups in preserving integrity of the joint region after cracking^48,49^.

One of the most significant findings of the study is the transition from the beam flexural-dominated failure observed in the control specimen to a more distributed flexure–shear interaction in the rubberized concrete specimens. This transition indicates that crumb rubber does not only act as a replacement material, but also modifies the internal stress field and the damage evolution mechanism within the joint region. From a structural safety perspective, progressive failure with visible cracking and gradual stiffness degradation is preferable to sudden brittle collapse because it provides warning and residual load-carrying capacity^50^.

Overall, the combined results demonstrate that rubberized concrete beam–column joints possess promising potential as sustainable structural components. Although the incorporation of crumb rubber resulted in a moderate reduction in peak load capacity and initial stiffness, these reductions were compensated by significant improvements in ductility, crack distribution, energy absorption, and failure stability. When combined with proper detailing, controlled axial load levels, and adequate transverse reinforcement, rubberized concrete joints can achieve reliable structural performance while simultaneously contributing to waste tire recycling and sustainable construction practice.

## Conclusions

Based on the experimental investigation, the validated three-dimensional nonlinear finite element analysis, and the parametric study conducted within the investigated ranges, the following conclusions can be drawn:

1. The structural performance of reinforced concrete beam–column joints was significantly influenced by the incorporation of crumb rubber as a partial replacement for fine aggregate. Although increasing the rubber content resulted in a gradual reduction in ultimate load capacity and initial stiffness, it considerably enhanced the deformation capacity, damage tolerance, and energy absorption of the joints.
2. The specimen containing 10% crumb rubber demonstrated the most balanced structural performance among the investigated mixtures, providing a favorable balance between strength, deformation capacity, and energy absorption while maintaining satisfactory load-carrying capacity. This replacement level represents an appropriate compromise between structural strength and deformation capacity.
3. The incorporation of crumb rubber promoted more distributed crack development and delayed damage localization, resulting in a more gradual failure process compared with the control specimen. All specimens exhibited beam flexural or flexure–shear dominant failure modes under monotonic loading.
4. The developed three-dimensional finite element model based on the Concrete Damage Plasticity (CDP) model successfully reproduced the experimental behavior, including the load–displacement response, crack development, and ultimate load capacity. The predicted peak loads agreed well with the experimental results, with errors ranging from 1.03% to 5.07%, confirming the reliability of the adopted numerical modeling approach.
5. The validated numerical model enabled an extensive parametric study that demonstrated the significant influence of the column axial load ratio, beam longitudinal reinforcement ratio, joint transverse reinforcement ratio, and concrete compressive strength on the structural behavior of beam–column joints.
6. Lower-to-moderate column axial load ratios provided the most favorable structural response by improving joint confinement, stiffness, and ultimate strength, whereas excessive axial compression reduced deformation capacity because of premature concrete crushing and accelerated post-peak strength degradation.
7. Increasing the beam longitudinal reinforcement ratio enhanced the ultimate load capacity and initial stiffness but reduced the displacement capacity of the joints, whereas increasing the joint transverse reinforcement ratio improved confinement effectiveness, crack control, structural stability, and post-peak performance.
8. Higher concrete compressive strength increased the load-carrying capacity and stiffness of the joints, whereas lower concrete strengths exhibited higher deformation capacity. Therefore, an intermediate concrete strength may provide a more balanced combination of strength and deformation capacity for rubberized concrete beam–column joints.
9. The findings of this study demonstrate that rubberized concrete can be considered a promising sustainable construction material for reinforced concrete beam–column joints, offering enhanced deformation capacity and a more progressive failure behavior while maintaining acceptable structural performance at appropriate crumb rubber replacement ratios. These findings support the potential application of rubberized concrete in structural members where improved damage tolerance and energy absorption are desirable.
10. The conclusions presented herein are limited to the investigated material properties, specimen geometry, reinforcement detailing, and monotonic loading conditions. Since reinforcement strain measurements and quantitative crack-width measurements were not included in the experimental program, the structural response was evaluated primarily through load–displacement behavior, crack patterns, and failure modes. Further experimental and numerical studies under cyclic and seismic loading are recommended to investigate cumulative damage, bond deterioration, hysteretic behavior, and long-term structural performance before extending the present findings to seismic design applications.

## Future work

- Expanding the experimental database to include a wider range of rubber replacement ratios, concrete strength grades, reinforcement detailing, and axial load levels in order to further validate and generalize the structural behavior of rubberized concrete beam–column joints.
- Investigating the performance of rubberized concrete beam–column joints under cyclic, reversed, and seismic loading conditions to assess hysteretic response, energy dissipation capacity, stiffness degradation, and long-term structural resilience.
- Studying time-dependent and durability-related effects such as creep, shrinkage, temperature variation, moisture exposure, and aggressive environmental conditions to evaluate their influence on bond characteristics, crack development, and service-life performance.
- Extending the validated numerical framework to interior joints, corner joints, multi-story frame systems, and full-scale structural applications, together with the development of performance-based design models and code-oriented recommendations for practical implementation.

## Data Availability

Availability of data and materials All data generated or analyzed during this study are included in this published article.
